# Dopamine release and dopamine-related gene expression in the amygdala are modulated by the gastrin-releasing peptide in opposite directions during stress-enhanced fear learning and extinction

**DOI:** 10.1038/s41380-024-02843-8

**Published:** 2024-11-23

**Authors:** Yoshikazu Morishita, Ileana Fuentes, Sofia Gonzalez-Salinas, John Favate, Jennifer Mejaes, Ko Zushida, Akinori Nishi, Charles Hevi, Noriko Goldsmith, Steve Buyske, Stephanie E. Sillivan, Courtney A. Miller, Eric R. Kandel, Shusaku Uchida, Premal Shah, Juan Marcos Alarcon, David J. Barker, Gleb P. Shumyatsky

**Affiliations:** 1 Department of Genetics, Rutgers University, Piscataway, NJ, USA.; 2 Endowed Department of Cognitive Function and Pathology, Institute of Brain Science, Nagoya City University Graduate School of Medical Sciences, Nagoya, Japan.; 3 Department of Psychology, Rutgers University, Piscataway, NJ, USA.; 4 Keck Center, Rutgers University, Piscataway, NJ, USA.; 5 Department of Statistics, Rutgers University, Piscataway, NJ, USA.; 6 Department of Molecular Medicine, The Scripps Research Institute, Jupiter, FL, USA.; 7 Howard Hughes Medical Institute, Columbia University, New York, NY, USA.; 8 Department of Integrative Anatomy, Nagoya City University Graduate School of Medical Sciences, Nagoya, Japan.; 9 Department of Pathology, Robert F. Furchgott Center for Neural and Behavioral Science, SUNY Downstate Health Sciences University, Brooklyn, NY, USA.

## Abstract

Fear extinction leads to a decrease of originally acquired fear responses after the threat is no longer present. Fear extinction is adaptive and critical for organism’s survival, but deficits in extinction may lead to exaggerated fear in animals or post-traumatic stress disorder (PTSD) in humans. Dopamine has recently emerged as essential for fear extinction and PTSD, however the neural circuits serving this dopamine function are only beginning to be investigated, and the dopamine intracellular signaling pathways are unknown. We generated *gastrin-releasing peptide* gene knockout (*Grp*^−/−^) mice and found that they exhibit enhanced fear memory in a stress-enhanced fear learning (SEFL) paradigm, which combines stress exposure and fear extinction, two features critical for developing PTSD. Using in vivo fiber photometry to record dopamine signals, we found that the susceptibility of *Grp*^*−/−*^ mice to SEFL is paralleled by an increase in basolateral amygdala (BLA) dopaminergic binding during fear conditioning and early extinction. Combined optogenetics and ex vivo electrophysiology showed an increase in presynaptic ventral tegmental area (VTA)- BLA connectivity in *Grp*^−/−^ mice, demonstrating a role of dysregulated input from the VTA on BLA function in the absence of the GRP. When examining gene transcription using RNA-seq and qPCR, we discovered concerted down-regulation in dopamine-related genes in the BLA of *Grp*^−/−^ mice following long-term SEFL memory recall that was not observed in naïve conditions. These experiments demonstrate that the GRP regulates dopamine function in stress-enhanced fear processing and identify the *Grp* as the first gene known to regulate dopaminergic control of fear extinction.

## INTRODUCTION

Animals and humans survive in the world by adapting their initial innate behavioral responses through learning to better navigate through the environment, which often includes an exposure to environmental threats [1, 2]. Animals perceive threat as innate or learned [3, 4]. Learned (conditioned) fear is acquired after a neutral conditioned stimulus (CS) becomes associated with an aversive innate (unconditioned) stimulus (US) [3, 5–8]. Fear conditioning in laboratory settings occurs quickly and requires only a few pairings of a CS with a shock US. Conditioned fear responses can also attenuate through the process called fear extinction. Fear extinction leads to a decrease of originally acquired fear responses after the threat is no longer present. During fear extinction in laboratory conditions, because the shock US is not present, the animal learns anew that the CS no longer predicts the US, and the conditioned fear response is suppressed [9]. Extinction does not erase the original fear memory, but rather creates a new memory representation, reflecting the more recent information that the CS no longer represents the threat. When environmental stress and threat are excessive, the organism may generate exaggerated and unbalanced neural responses, often leading to prolonged fear [10, 11]. While extinction is normally adaptive, its deficiency may lead to excessive fear in animals, mirroring pathological responses in humans.

Fear extinction is dependent on the interactions of several brain regions: the amygdala, hippocampus, nucleus accumbens (NAc), medial prefrontal cortex (mPFC) and ventral tegmental area (VTA) among others [10]. Deficits in fear extinction are a major contributor to posttraumatic stress disorder (PTSD) in humans [12]. Importantly, PTSD is one of the more tractable mental disorders, genetically and behaviorally, as it can be studied using rodent models of impaired fear extinction [13, 14]. There has been a significant effort to examine the neural circuits involved in fear extinction and PTSD, but the molecular identity and organizational logic of cell types that lie at the core of these processes have still not yet been identified [11, 13, 15–17].

In addition to the well-established role of the dopaminergic system in reward and appetitive learning, accumulating recent evidence strongly suggests dopamine importance in fear extinction learning: dopamine was shown to provide the signal of the omission of expected aversive US in several species, including flies, rodents and humans [18–34]. Dopamine neurons interface with fear circuits through VTA and substantia nigra (SNc) projections to the amygdala [35, 36]. Nonetheless, we still have very little information about how dopaminergic inputs to the amygdala process fear extinction, nor do we know which specific cell populations or molecular pathways are involved [37].

To begin investigating molecules and cells that may regulate dopamine function in the basolateral amygdala (BLA) in relationship to fear extinction, we focused on the *gastrin-releasing peptide* (*Grp*) gene, which is enriched in a specific population of the excitatory neurons of the BLA [38], as well as in several key inputs and outputs to the amygdala, including the VTA [39] where it colocalizes almost exclusively with markers of dopaminergic neurons [39, 40]. Moreover, dopamine and the GRP have opposite effects on the inhibition of conditioned fear – the GRP enhances BLA inhibition, while dopamine suppresses BLA inhibition [38, 41, 42]. In this study, we investigated a potential role of BLA and VTA GRPergic (GRP + ) neurons in fear extinction using a Stress-Enhanced Fear Learning (SEFL) paradigm [10, 43, 44] and the *Grp* knockout (KO; *Grp*^−/−^) mice. We found that in the absence of the GRP, dopamine release is enhanced during fear learning and early phases of extinction, however dopamine-related gene transcription is decreased following fear extinction recall during later phases. Our results establish the GRP as one of the first molecules known to regulate dopamine control of fear extinction.

## MATERIALS AND METHODS

The detailed methods for the mice, immunohistochemistry, rAAV2-based retrograde tracing, stereotaxic surgery, fear conditioning, anxiety assays, bombesin-saporin injections, SEFL, dLight fiber photometry, ex vivo electrophysiology, D1 antagonist, RNA isolation, qRT-PCR, Western blotting and transcriptome analysis can be found in Supplementary Materials and Methods.

## RESULTS

### Generation of the *Grp*^−/−^ mice and mapping of the GRPergic neural circuitry

**Fig. 1:**
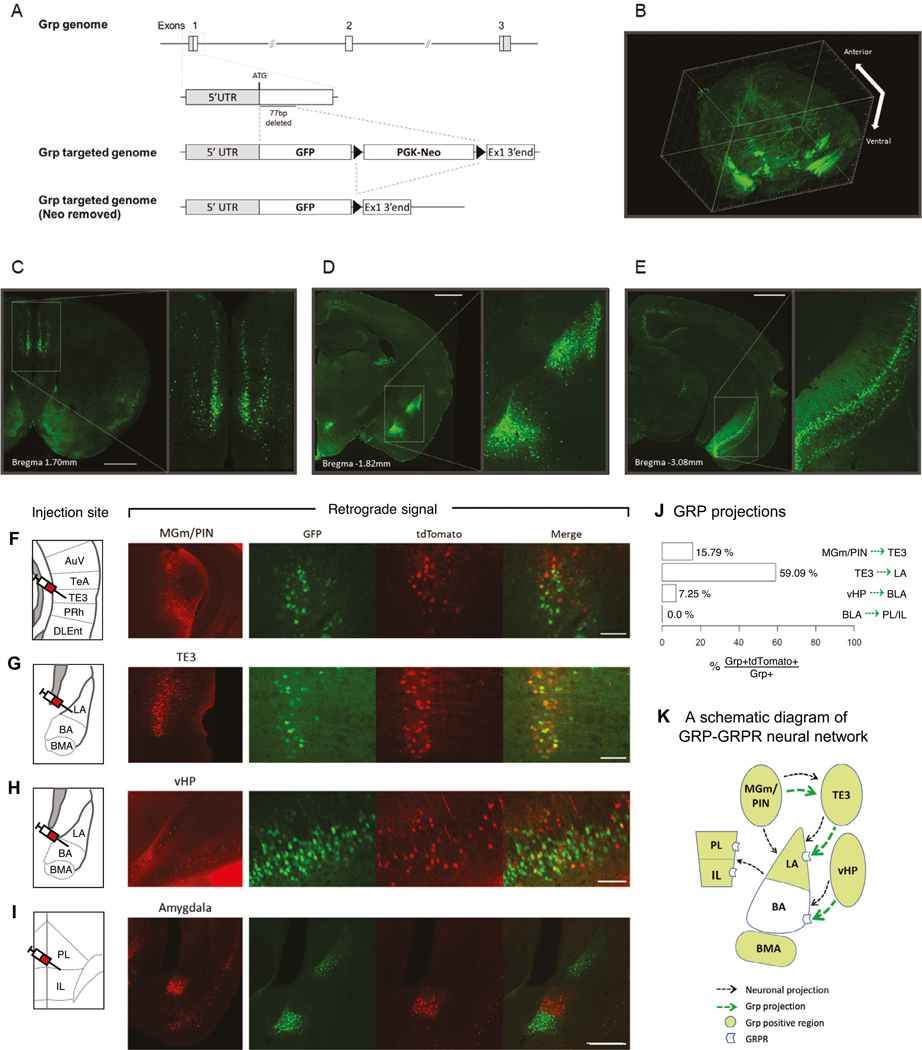
The generation of the *Grp*^*−/−*^ mouse and retrograde tracing of the GRPergic projections. **A** Schematic diagram of the *Grp* gene knockout. Part of exon 1 of the *Grp* gene (77 bp from translation start site) was removed, and GFP ORF and neomycin cassette were inserted. The neomycin cassette was later removed by FLP-mediated excision in vivo. **B** The 3-dimensional map of the GFP signal in the *Grp*^*−/−*^ mouse brain. The GFP is mainly expressed in the mPFC (**C**), basolateral amygdala (**D**) and ventral hippocampus (**E**). rAAV2-retro-CaMKII-tdTomato was injected into (**F**) the TE3 area of the auditory cortex, (**G**) lateral nucleus of the amygdala (LA), (**H**) basal nucleus of the amygdala (BA) or (**I**) mPFC of the *Grp*^*−/−*^ mice. Three weeks following injections, the mice were perfused, and the brains were coronally sectioned at a thickness of 40 μm. The left panel shows the site of injection. Representative figures illustrate the retrograde labeling (red) of the GRPergic neurons (green) from each injection site. **J** Percentage of the GRP-positive cells expressing the retrograde tracer (*rAAV2-retro-CaMKII-tdTomato*) within the BLA-associated neural circuitry. **K** Schematic diagram of GRPergic projections within the BLA-associated neural circuitry.

We developed the *Grp*^−/−^ mice in which the majority of exon 1 of the *Grp* gene was deleted and replaced with the green fluorescent protein (GFP) (Fig. 1A; Supplementary Fig. 1A–C). The *Grp*^−/−^ mice of both sexes develop normally and show no gross abnormalities throughout the body, including the brain (Supplementary Fig. 1D). Staining with the anti-GFP antibody showed that the GFP is highly enriched in the lateral (LA) and basomedial (BMA), but not basal (BA), nuclei of the amygdala as well as in the ventral CA1 area of the hippocampus and mPFC (Fig. 1B–E). In addition, we observed GFP expression in the retrosplenial cortex, dorsal subiculum, auditory cortex, and the entorhinal/perirhinal cortex, similar to the endogenous *Grp* expression previously examined by RNA in situ hybridization [38, 45]. Therefore, expression of the GFP knocked-in in exon 1 of the *Grp* gene is very similar to the endogenous GRP expression.

To map the GRPergic neurons onto the amygdala-associated neural circuitry, we injected retrograde neuronal tracer rAAV2-retro-CaMKII-tdTomato (rAAV2) [46] in several brain regions in *Grp*^−/−^ mice. When rAAV2 was injected in the TE3 area, the retrograde labeling co-localized with the GRP-positive cells in the MGm/PIN area of the auditory thalamus (Fig. 1F). When rAAV2 was injected in the lateral nucleus of the amygdala (LA), the areas labeled with tdTomato were the TE3 area of the auditory cortex (Fig. 1G) and the MGm/PIN (Supplementary Fig. 2), two major regions sending projections to the LA [3, 47]. However, only the TE3 area showed co-localization of the tdTomato and GFP (Fig. 1G); the MGm/PIN area had no GRP-positive cells co-labeled with the tdTomato (Supplementary Fig. 2B). The results of the injections into TE3 and LA show that GRP-positive cells project to the LA via the indirect MGm/PIN- > TE3-LA pathway but not via the direct MGm/PIN- > LA pathway.

When rAAV2 was injected in the basal nucleus of the amygdala (BA), the co-localization of tdTomato and GFP was observed in the ventral hippocampus (vHP; Fig. 1H), the major region projecting to the BA [48].

To examine the BLA-mPFC projections, we injected rAAV2 in the mPFC. Interestingly, most of the BLA projections originated from the BA area, which by itself is GRP-negative but is located exactly between the two major GRP-positive amygdala areas, the LA and the basomedial nucleus of the amygdala (BMA). There was no co-localization between GRP-positive cells and those labeled with tdTomato in the BA area or any other areas of the amygdala projecting to the mPFC (Fig. 1I). Thus, the BLA GRPergic neurons do not project to the mPFC. The BA however receives the major dopaminergic projections from thè ventral tegmental area (VTA) neurons, some of which are GRP-positive (see below the section about the GRPergic cells in the VTA). Quantification of these connections showed a higher density of GRP-positive cells in the TE3- > LA projections followed by the MGm/PIN - > TE3 and vHP- >BLA projections (Fig. 1J). These results show that the GRP expression maps a very specific neural circuitry entering the amygdala and may play a unique role in processing of fear-related sensory information (Fig. 1K).

### The *Grp*^−/−^ mice show an enhancement in cued and contextual long-term fear memory

**Fig. 2:**
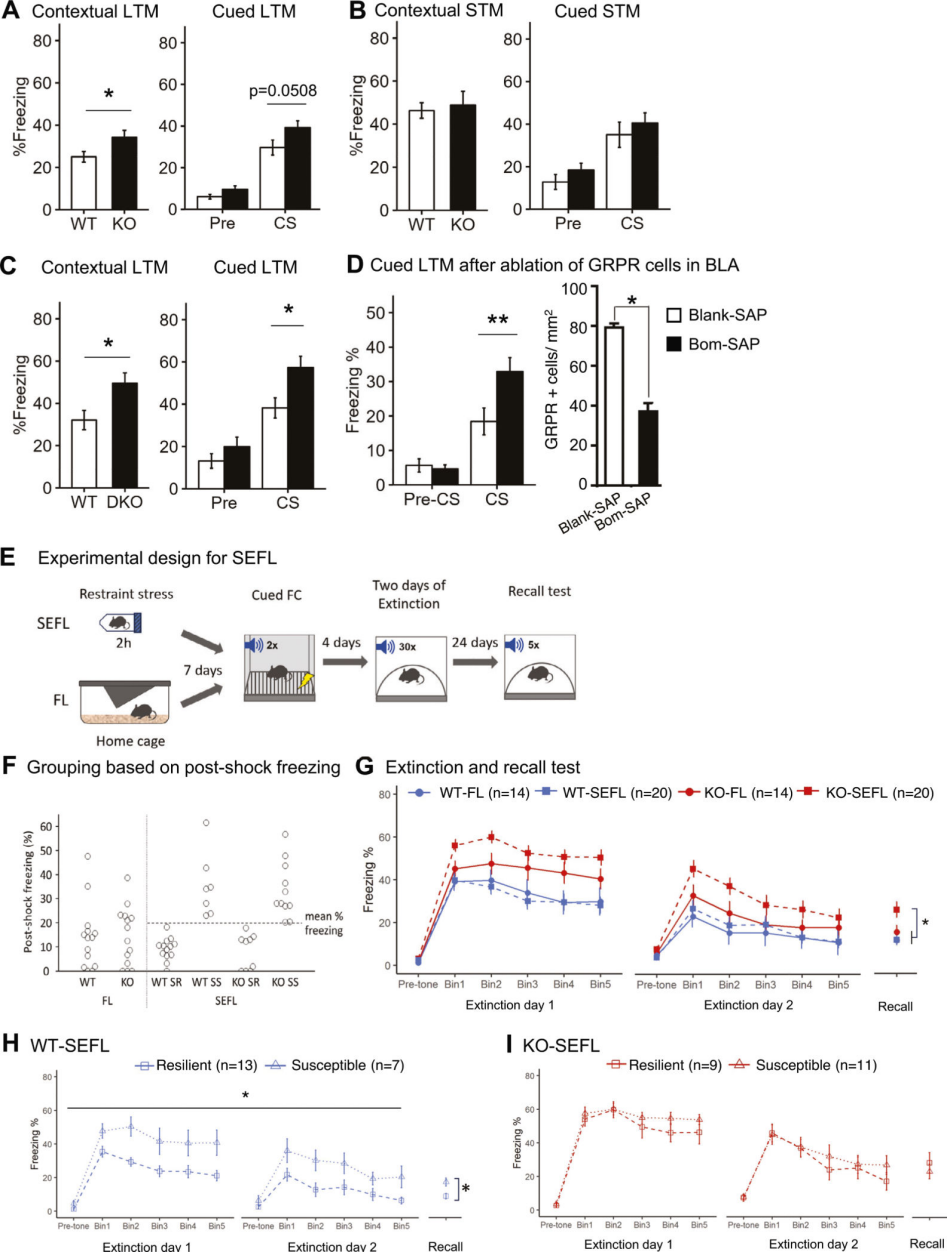
The *Grp*^−/−^ mice exhibit an enhancement in long-term fear memory and an increased susceptibility in stress-enhanced fear learning (SEFL). Mice were placed individually in a conditioning chamber for 120 s, and a tone was applied for 30 s that co-terminated with a foot shock (0.75 mA, 2 s). After an additional 30 s in the chamber, the mice were returned to their home cage. 24 h after training (test for long-term memory, LTM), the mice were placed back into the chamber for 180 s (contextual test). 3 h after the contextual test, the mice were placed into a novel environment, and 60 s later the tone was applied for 180 s (cued test). **A** The *Grp*^−/−^ mice showed significant enhancement of contextual LTM (*p* = 0.031). A Two-way ANOVA of freezing during cued LTM showed differences in Genotype and between Pre-CS and CS but the interaction was not significant (Genotype F(1,80) = 6.64, *p* = 0.011; Stimulus F(1,80) = 25.4, *p* < 0.001; Genotype X Stimulus F(1, 80) = 1.43, *p* = 0.23). A comparison of freezing between WT and *Grp*^−/−^ mice showed *p* = 0.0508 for the CS only and *p* = 0.104 for the Pre-CS (WT *n* = 21, *Grp*^−/−^ *n* = 22). **B** Contextual short-term memory (STM) and cued-STM were unaffected in *Grp*^−/−^ mice (Contextual-STM: *p* = 0.722, WT *n* = 10, *Grp*^−/−^ *n* = 9; Cued-STM: *p* = 0.115, WT *n* = 12, *Grp*^−/−^ *n* = 13). **C** GRP/GRPR double knockout mice showed significant enhancement of contextual and cued LTM (WT *n* = 10, DKO *n* = 10, contextual LTM *p* = 0.018, cued LTM *p* = 0.015). *p < 0.05, unpaired Student’s *t*-test. Data presented as mean ± SEM. **D** Ablation of GRPR cells in the BLA of WT mice increased freezing during long-term memory test of cued fear conditioning (Two-way ANOVA: Treatment X Stimulus: F(1, 58) = 6.45, *p* = 0.0138; Tukey post-hoc test ** *p* = 0.0062). The number of GRPR cells was significantly decreased in Bom-SAP mice (*p < 0.01, t-test). Blank-SAP *n* = 16, Bom-SAP *n* = 16. **E** An overview of the SEFL paradigm. WT and *Grp*^−/−^ mice were assigned to SEFL or FL (fear learning) groups. Mice in SEFL were subjected to 2 h of restraint stress while mice in FL were gently handled in home-cage. Training in cued fear conditioning (Cued-FC) took place seven days after. Extinction training (4 days following during fear conditioning) and remote memory retrieval tests (30 days post-shock) were performed in a novel context. **F** Post-shock freezing (last 30 sec during fear conditioning) is used to separate the subjects into the susceptible and resilient groups. Animals that froze above the mean % freezing for the stressed group were classified as stress-susceptible (SS), while those that fell below the mean were classified as stress-resilient (SR). **G** Course of extinction and recall test in SEFL. Shown are five bins (6 tones in each bin) of the conditioned stimulus (CS) presentations during extinction (WT-FL *n* = 14, WT-SEFL *n* = 20, KO-FL *n* = 14, KO-SEFL *n* = 20; Genotype F_1,64_ = 18.137, *p* < 0.001, Stress F_1,64_ = 1.918, *p* = 0.171, Genotype X Stress F_1,64_ = 2.287, *p* = 0.135). In the recall test, a significant interaction between the effect of Genotype and Stress on freezing was observed in the SEFL group (Genotype F_1,64_ = 15.119, *p* < 0.001, Stress F_1,64_ = 4.667, *p* = 0.0345, Genotype X Stress F_1,64_ = 4.269, *p* = 0.042). **H, I** Stressed and fear-conditioned (SEFL) mice can be separated into two subgroups, stress resilient (SR) or stress susceptible (SS), based on their post-shock freezing during fear conditioning. Their extinction profiles showed significant difference between SR and SS in extinction and recall test in WT-SEFL but not in KO-SEFL (WT-SEFL-SR *n* = 13, WT-SEFL-SS *n* = 7, KO-SEFL-SR *n* = 9, KO-SEFL-SS *n* = 11; WT-SEFL extinction: *Susceptibility F_1,18_ = 11.380, *p* = 0.003, *Recall: *p* = 0.008; KO-SEFL extinction: Susceptibility F_1,18_ = 1.711, *p* = 0.206, Recall: *p* = 0.698). Behavioral analysis was performed using two-way repeated-measures ANOVA. Data presented as mean ± SEM.

To characterize memory of the *Grp*^−/−^ mice, we examined them in the standard protocol of fear conditioning. Post-shock freezing during training was not different between genotypes (Supplementary Fig. 3A). *Grp*^−/−^ mice showed stronger long-term memory (LTM) in both cued and contextual fear (Fig. 2A). We also analyzed *Grp*^−/−^ mice for short-term memory (STM) testing them 1 hour after training using independent groups of *Grp*^−/−^ mice. There was no significant difference between wildtype and *Grp*^−/−^ mice in both cued and contextual STM (Fig. 2B and Supplementary Fig. 3B, C). Thus, the enhancement in memory observed in *Grp*^−/−^ mice is specific to long-term, but not short-term, fear memory, demonstrating the specificity of the GRP/GRPR signaling pathway for long-term memory related to fear [38].

Next, we crossed the *Grp*^−/−^ mice (GRP KO) with GRPR KO mice and assessed GRP/GRPR double KO mice in fear conditioning. The double KO mice showed enhanced long-term contextual and cued fear memory assessed 24 h after fear conditioning (Fig. 2C and Supplementary Fig. 3D). The fact that the double KO animals have stronger fear memory than the *Grp*^−/−^ mice suggests that the GRP and GRPR may have other, perhaps secondary, ligands/receptors that they might bind, such as other members of the bombesin family [49].

To rule out possible developmental effects of removing GRP/GRPR system early in life and determine the specific effects of GPR signaling in the amygdala, we selectively ablated interneurons expressing GRPR using saporin-based cell elimination in the BLA of wildtype mice at the adult stage (Fig. 2D) [50]. Mice injected with bombesin-saporin (bombesin is a frog peptide related to the GRP with high affinity to the GRPR [51]) showed a ~ 50% reduction in GRPR-expressing cells and had significantly higher freezing compared to mice injected with control blank-saporin in cued fear conditioning. Therefore, potential developmental changes are not likely to be responsible for the enhanced fear memory phenotype in GRP or GRPR KO mice.

### Enhanced amygdala activity of the *Grp*^−/−^ mice following fear conditioning

Based on our observations that GPR-related knockouts enhance fear memory, we hypothesized that the absence of GRP or GRPR may elevate neural activity in the amygdala. Expression of immediate-early genes (IEG) is activity-dependent and often used as an indicator of neural activity. Quantitative real-time PCR (qPCR) revealed that RNA expression of IEG *c-Fos* and *Arc* was increased in the BLA of *Grp*^−/−^ mice compared to their wildtype counterparts 30 min after single-paired cued fear conditioning (Supplementary Fig. 4). Activity of *c-Fos* and *Arc* was normal in naïve *Grp*^−/−^ mice. These results suggest that the neural activity in the amygdala of *Grp*^−/−^ mice is enhanced after fear conditioning, which is consistent with the enhanced memory *Grp*^−/−^ mice display in fear conditioning.

### *Grp*^−/−^ mice show normal anxiety and pain sensitivity

We examined anxiety levels of the *Grp*^−/−^ mice using the elevated plus maze (EPM), open field (OF) and light-dark (LD) box (Supplementary Fig. 5A–C). The statistical analysis showed that there was no significant difference between the genotypes in all three tests. To verify that the increase in freezing displayed by the *Grp*^−/−^ mice in fear conditioning was not due to an increased sensitivity to the shock, we examined their pain sensitivity by movement, vocalization and jump (Supplementary Fig. 5D). There was no difference between genotypes in the intensity of the shock required to elicit these three behaviors. We interpret these findings to indicate that the increase in freezing observed in fear conditioning is due to differences in memory, but not in anxiety or pain sensitivity, confirming the behavioral phenotype of GRPR KO mice [38].

### *Grp*^−/−^ mice exhibit increased susceptibility to stress-enhanced fear learning (SEFL)

To examine a possible role of the GRP in linking stress and fear, we examined the *Grp*^−/−^ mice in a behavioral task where mild stress precedes fear conditioning and fear extinction, using SEFL (Fig. 2E) [52]. Each genotype had two groups of mice: one group (SEFL) was exposed to 2 h of restraint stress, while another group – designated only for fear learning (FL) – was kept naïve in a home cage. Seven days later all mouse groups were trained in fear conditioning (Cued FC; Fig. 2E, F) using two tone-shock pairings. Four days after fear conditioning, all groups underwent two days of fear extinction, and recall memory was tested after additional 24 days. A repeated-measures three-way ANOVA revealed the significant main effect of the genotype on the freezing during extinction (Fig. 2G). In the recall test, a significant interaction between the effect of genotype and stress on freezing was observed in the SEFL group. Freezing during recall was higher in the KO-SEFL group compared to other groups (*p* = 0.0196 vs KO-FL; *p* = 0.0003 vs WT-SEFL; *p* = 0.001 vs WT-FL, Tukey post-hoc test). We calculated the percent of decrease in freezing by subtracting freezing in bin 5 from freezing in bin 1 for each extinction session and found that the extinction rate was similar between the genotype or stress groups in Extinction 1 or 2 (Supplementary Fig. 6 A, B). We also calculated the percent of increase in freezing during Recall by subtracting the freezing during the last bin of Extinction 2 from freezing during Recall and found no significant differences in the increase of freezing between the groups (Supplementary Fig. 6C).

Following fear conditioning, the stressed group was separated into two subgroups, resilient and susceptible, based on their freezing performance during one minute of post-shock freezing (Fig. 2F), as this measure is used as an index of stress susceptibility in the SEFL protocol [52]. Animals that froze above the mean percent freezing in the stressed group were classified as stress-susceptible (SS), while those that fell below the mean were classified as stress-resilient (SR). The ratio of the number of mice in the susceptible group to the resilient group was higher in the *Grp*^−/−^ mice compared to wildtype mice. There was also a significant difference in freezing during extinction and recall between the resilient and susceptible subgroups in wildtype mice (Fig. 2H). However, there was no significant difference between the knockout stress-resilient group and knockout stress-susceptible group in extinction and recall (Fig. 2I). A comparison between Genotype and Susceptibility factors revealed that the KO mice, regardless of being stress-resilient or stress-susceptible, showed higher freezing compared to the WT mice (statistical differences are in Genotype but not in the interaction of Genotype X Susceptibility; Supplementary Fig. 7). The results suggest that the deletion of the *Grp* gene confers susceptibility to stress combined with conditioned fear.

### Downregulation of the dopamine-signaling pathway in the amygdala of the *Grp*^−/−^ mice following SEFL recall

**Fig. 3:**
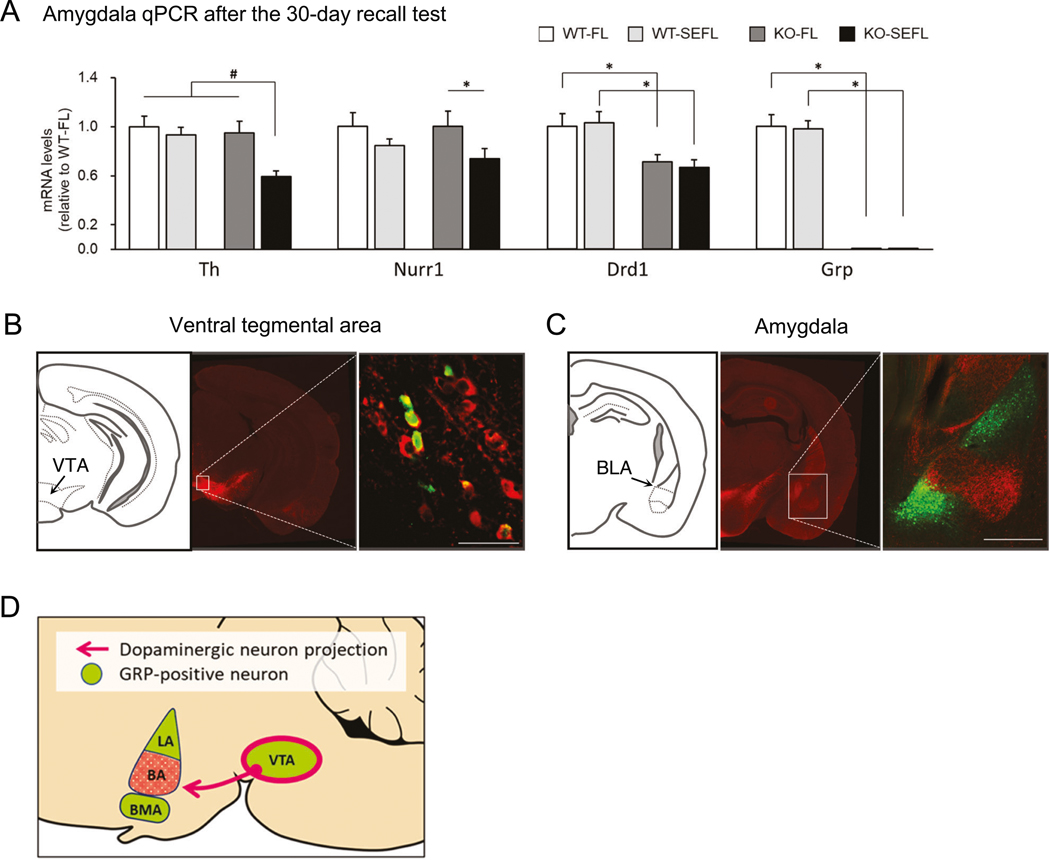
Dopamine signaling-related genes are downregulated in *Grp*^*−/−*^ mice following recall in SEFL. **A** Quantitative real-time PCR (qPCR) analysis of dopamine signaling-related genes that were differentially expressed in the BLA (WT-FL *n* = 12, WT-SEFL *n* = 18, KO-FL *n* = 13, KO-SEFL *n* = 18). After the recall test, the mice were returned to their home cage and the amygdala tissue was dissected 30 min later. All target mRNA expression levels were normalized to *Gapdh* expression and verified by normalization to *β-actin*. Results are expressed as x-fold change normalized to wildtype controls. All measurements were performed in triplicate. Two-way ANOVA interaction effect #*p* < 0.05. Bonferroni test *p < 0.05. Data presented as mean ± SEM. **B, D** The brains were harvested from 3-month-old *Grp*^*−/−*^ male mice and coronally sectioned at a thickness of 40 μm. Immunohistochemistry was performed with antibodies against the tyrosine hydroxylase (TH, red) and GFP (green). **B** The GRP is expressed within dopaminergic subpopulations of the VTA. **C** Dopaminergic neurons project to the BA subregion of amygdala. GRPergic cells in the LA and BMA are not receiving projections from the dopaminergic VTA neurons. **D** Schematic of the dopaminergic projections from the VTA to the GRP-positive regions.

We used qPCR to examine transcription of several genes known to be induced following SEFL recall [52], PTSD or stress [10, 53]. We found that several genes involved in the dopamine signaling were downregulated in the basolateral amygdala of the *Grp*^−/−^ mice (KO-SEFL) compared to control mice following SEFL memory recall (Fig. 3A and Supplementary Fig. 8). One of these genes encodes for the tyrosine hydroxylase (TH). There was a significant interaction between the effect of genotype and stress on a decrease in expression of the *Th* mRNA as shown by two-way ANOVA (Fig. 3A and Table S1). To verify that the observed *Th* mRNA was trafficked from the VTA cell nuclei to the BLA synapses to be translated into TH protein there, we examined TH protein expression. Western analysis showed similar levels of the TH protein between *Grp*^−/−^ and wildtype mice in naïve conditions in the hippocampus (Supplementary Fig. 9A). Thus, *Th* mRNA and TH protein expression in the BLA and hippocampus confirm previous evidence that the *Th* mRNA is trafficked and locally translated at synapses [54, 55].

Other dopamine-related genes found in our qPCR analysis include nuclear receptor related 1 protein (NURR1) and dopamine receptor D1 (DRD1). The *Drd1* mRNA was also decreased in the *Grp*^−/−^ mice that received fear extinction but no stress (FL group). A two-way ANOVA followed by post-hoc test also revealed a significant acute stress-dependent decrease of *Nurr1* mRNA expression in the *Grp*^−/−^ mice and GRP knockout-dependent decrease of *Drd1* mRNA expression (Fig. 3A and Table S1). qPCR analysis revealed no difference in mRNA expression of the *Th*, *Nurr1*, *Drd1* and *Drd2* genes in the VTA and BLA of naïve wildtype and *Grp*^−/−^ mice (Supplementary Fig. 9B, C). These results suggest the possibility that the changes in expression of dopamine-related genes found after recall were induced in the *Grp*^−/−^ mice by long-term memory retrieval during SEFL.

### Co-expression of the GRP and tyrosine hydroxylase in the ventral tegmental area

To investigate the relationship between the GRPergic and dopaminergic neurons, we performed co-immunostaining for the TH and *Grp*-promoter-driven GFP on brain sections of the *Grp*^*−/−*^ mice. TH immunostaining showed expression in the fibers in the BLA and in cell bodies in the VTA, confirming previous work showing that the TH is expressed in the VTA, which sends projections to the amygdala (Fig. 3B, C) [56]. In the VTA, we found that the GFP and TH overlap. Combined, our results suggest that a certain proportion of the VTA neurons, are positive for both dopamine and GRP, and that TH- and GRP-positive fibers are present in the BLA (Fig. 3D).

### Enhanced fear is supported by differences in dopamine signaling

**Fig. 4:**
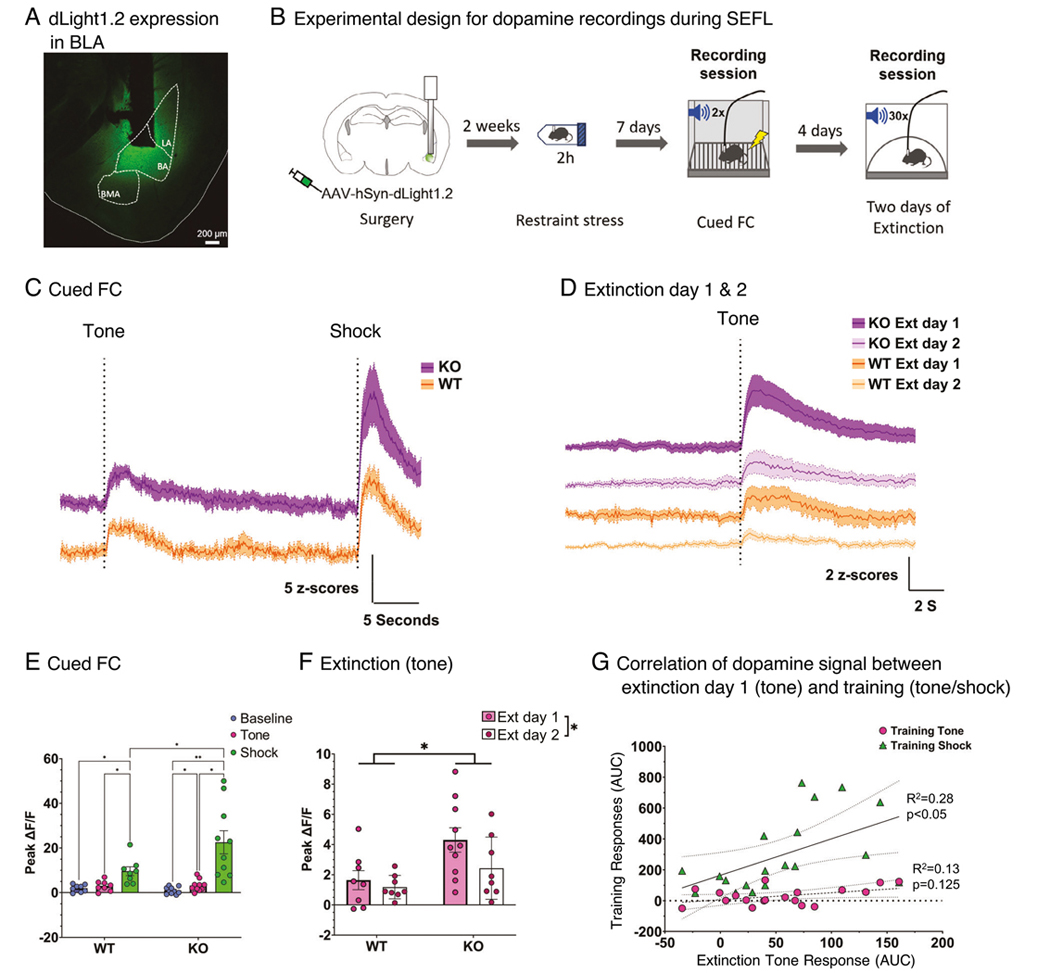
GRP knockout mice show stronger dopamine responses to shock and learned fear cues. **A** The fluorescent dopamine sensor dLight was injected into the BA and an optic fiber was implanted for recording dLight signals (**B**) Mice were run using a SEFL protocol and dLight responses were recorded during fear learning and subsequent extinctions sessions. increases in dopamine were observed to the tone and shock during fear learning (**C**) as well as to the shock-paired tone during extinction (**D**). **E** dLight responses to the shock were larger in the KO group than in the WT group during training. Baseline: *p* = 0.963, Tone: *p* = 0.963, Shock: *p* = 0.0187. Group x Epoch: F(2,20) = 3.515, *p* = 0.0492. Post-hoc holm-sidak for Shock (KO vs. WT): *p* = 0.0187. **F** dLight responses to the shock-paired tone were also larger during the first extinction session. Extinction 1 Tone: *p* = 0.0439, Extinction 2 Tone: *p* = 0.7228. Group x Session: F(1,10) = 7.638, *p* = 0.0020. Post-hoc holm-sidak for Ext 1 Tone (KO vs. WT): *p* = 0.0439. **G** Responses to the tone during extinction were directly related to the magnitude of the dLight response to the shock during training. Shock: R^2^ = 0.70, *p* < 0.001, Tone: R^2^ = 0.096, *p* = 0.33.

To determine whether changes in dopaminergic signaling might contribute to the modulation of fear memories, we recorded dopamine responses during SEFL training using the fluorescent dopamine sensor dLight1.2 (Fig. 4A, B). We observed that both the *Grp*^−/−^ and wildtype mice showed increased dLight responses to the tone and shock during fear learning (Main effect of event: F(2,49) = 19.47, *p* < 0.0001; Fig. 4C), that carried forward to the tone during extinction (Fig. 4D). The dLight responses to the shock were greater in both the *Grp*^−/−^ and wildtype mice (*Grp*^−/−^: *p* = 0.009; WT: *p* = 0.016). However, responses to the tone were only greater in the *Grp*^−/−^ mice when compared to baseline (*p* = 0.03). Additionally, dLight responses to the shock were greater in the *Grp*^−/−^ mice during fear learning than those observed in wildtype mice (Group x Event interaction: F(2, 48) = 4.60, *p* < 0.05; *Grp*^−/−^ vs. WT Shock: *p* = 0.0007; Fig. 4E), and subsequently greater to the tone during the first extinction session (Main effect of Group: F(1, 16) = 6.054, *p* = 0.026; Fig. 4F). Moreover, the magnitude of the dLight response to the tone during extinction was directly related to the magnitude of the dLight response to the shock during learning, but not to that of the tone during fear learning (Fig. 4G). Analysis of the behavior during photometry recordings showed that post-shock freezing was not different between genotypes, and that the ratio of the susceptible group to the resilient group was higher in the *Grp*^−/−^ mice compared to wildtype mice (Supplementary Fig. 10A, B). Freezing during extinction was not different between genotypes, most likely because of changes in mobility due to the implanted optic fibers (Supplementary Fig. 10C). When we included calculations of within-subject changes, the increased signal observed in the *Grp*^−/−^ mice during training and extinction was observed as well (Supplementary Fig. 10D, E). Thus, the observed differences in dopamine responses during SEFL are driven by genotype (GRP KO vs. WT) differences. From these results, we infer that dopamine signaling is important for regulating the initial response to a noxious stimulus, and that amplified responses to the cue and stimulus during training may be critical for determining subsequent susceptibility to learned fear cues.

### Occluded presynaptic VTA-BLA connectivity in the *Grp*^−/−^ mice

**Fig. 5:**
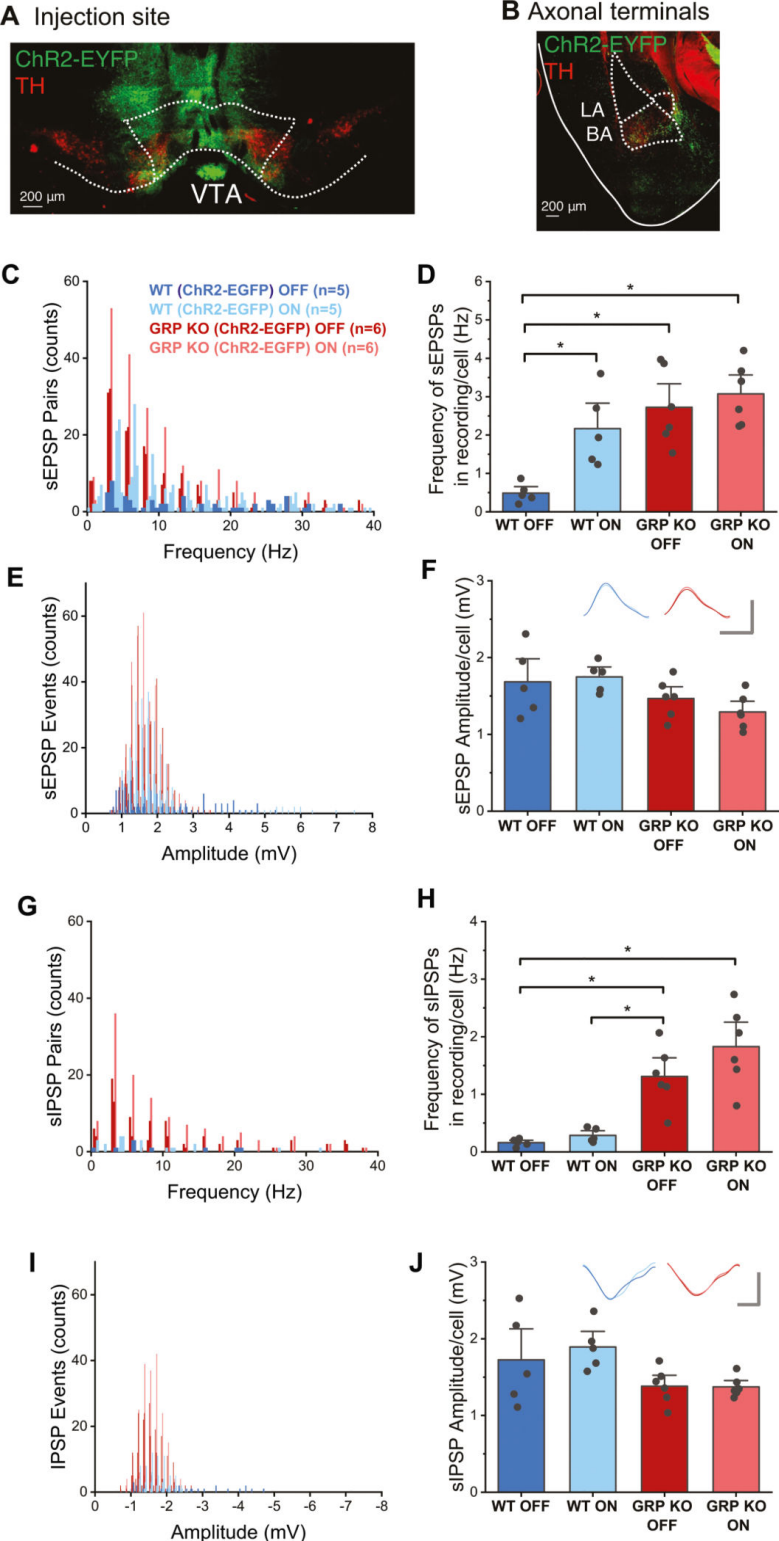
The VTA-BLA synaptic connection has a presynaptic excitatory dysregulation in the GRP KO mice. **A** Representative image of AAV-hSyn-ChR2-EYFP injection in the VTA. Immunohistochemistry against TH shows that the injection site included dopaminergic cells. **B** VTA-projecting axons in the BA were revealed by immunohistochemistry against GFP; these axons overlapped with TH expression. LA=lateral amygdala. **C** Frequency histogram of sEPSPs recorded from BLA neurons in WT and GRP KO mice before and during optogenetic stimulation of VTA-BLA terminals. **D** Average frequency of sEPSP events in recorded BLA neurons. Two-way ANOVA, Genotype F(1,21) = 19.49, *p* = 0.0003; Optogenetic F(1, 21) = 8.14, *p* = 0.010; Genotype X Optogenetic F(1, 21) = 3.49, *p* = 0.078. *Tukey: WT OFF × WT ON, *p* = 0.023; WT OFF x GRP KO OFF, *p* = 0.0016; WT OFF × GRP KO ON, *p* = 0.0004. All other comparisons *p* > 0.05. **E** Amplitude histogram of sEPSPs recorded from BLA neurons in WT and GRP KO mice before and during optogenetic stimulation of VTA-BLA terminals. **F** Average amplitude of sEPSP events in recorded BLA neurons. Inserts, averaged sEPSPs representing each condition; scale bars: 1 mV and 10 ms. Two-way ANOVA, Genotype F(1,21) = 7.22, *p* = 0.01; Optogenetic F(1, 21) = 0.19, *p* = 0.66; Genotype X Optogenetic F(1, 21) = 2.81, *p* = 0.06. All Tukey comparisons *p* > 0.05. **G** Frequency histogram of sIPSPs recorded from BLA neurons in WT and GRP KO mice before and during optogenetic stimulation of VTA-BLA terminals. **H** Average frequency of sIPSPs in recorded BLA neurons. Two-way ANOVA, Genotype F(1,21) = 46.09, *p* = 2.3 × 10^−6^; Optogenetic F(1, 21) = 2.63, *p* = 0.12; Genotype × Optogenetic F(1, 21) = 0.96, *p* = 0.34. *Tukey: WT OFF x GRP KO OFF, *p* = 0.0034; WT OFF x GRP KO ON, *p* = 6.8 × 10^−5^; WT ON x GRP KO OFF, *p* = 0.009; WT ON x GRP KO ON, *p* = 1.73 × 10^−4^. All other comparisons *p* > 0.05. **I** Amplitude histogram of sIPSPs recorded from BLA neurons in WT and GRP KO mice before and during optogenetic stimulation of VTA-BLA terminals. **J** Average amplitude of sIPSP events in recorded BLA neurons. Inserts, averaged sIPSPs representing each condition; scale bars: 1 mV and 10 ms. Two-way ANOVA, Genotype F(1,21) = 8.36, *p* = 0.009; Optogenetic F(1, 21) = 0.28, *p* = 0.60; Genotype X Optogenetic F(1, 21) = 2.98, *p* = 0.06. All Tukey comparisons *p* > 0.05.

To examine functional connectivity between the VTA and BLA, we injected the VTA of the *Grp*^−/−^ and WT mice with the *AAV-hSyn-ChR2-EGFP* (Fig. 5A, B) and examined the properties of spontaneous EPSPs (sEPSPs) and IPSPs (sIPSPs) in BLA neurons during optogenetic stimulation of VTA-BLA projecting axons. Light stimulation increased the frequency of sEPSPs in the WT mice (Fig. 5C, D). Notably, the frequency of sEPSPs observed in *Grp*^−/−^ mice at baseline conditions (without light stimulation) matched that of the WT mice under light stimulation and did not significantly change under light stimulation (Fig. 5C, D). We found similar results with the number of sEPSPs (Supplementary Fig. 11A). In contrast, sEPSP amplitude did not significantly change in WT or *Grp*^−/−^ mice under light stimulation, nor sEPSP amplitude was different between the groups (Fig. 5E, F). Similarly, the median sEPSP frequency in WT or *Grp*^−/−^ mice remained unchanged with light stimulation, although the *Grp*^−/−^ mice showed significantly lower median sEPSP frequency than the WT mice at baseline conditions (Supplementary Fig. 11B). Light stimulation did not significantly change sIPSP frequency in the WT mice, although the *Grp*^−/−^ mice showed a significantly heightened frequency of sIPSPs at baseline compared to WT neurons at baseline, which did not significantly change with light stimulation (Fig. 5G, H). Light stimulation did not significantly affect the amplitude of sIPSPs in WT or *Grp*^−/−^ mice, nor was sIPSP amplitude significantly different between the groups (Fig. 5I, J). Similar results were found with the analysis of the number of sIPSPs (Supplementary Fig. 11C) and median sIPSP frequency (Supplementary Fig. 11D). In addition to the optogenetic studies, we found a 4 mV more depolarized resting membrane potential and increased spike activity in the *Grp*^−/−^ compared to WT mice (Supplementary Fig. 11E, F). Our data, showing strong dopaminergic VTA-BLA projections (Fig. 3B, D), confirm previous research that indicates that the majority ( > 95%) of the VTA-BLA projections are dopaminergic [56–58]. Therefore, our results, showing occluded presynaptic VTA-BLA connectivity in the *Grp*^*−/−*^ mice, demonstrate a possible role of dysregulated dopaminergic input from the VTA on BLA function in the absence of the GRP.

### RNA-sequencing confirms and extends the qPCR data on the number of genes in the dopamine-signaling pathway with decreased expression in the *Grp*^−/−^ mice following SEFL recall

**Fig. 6:**
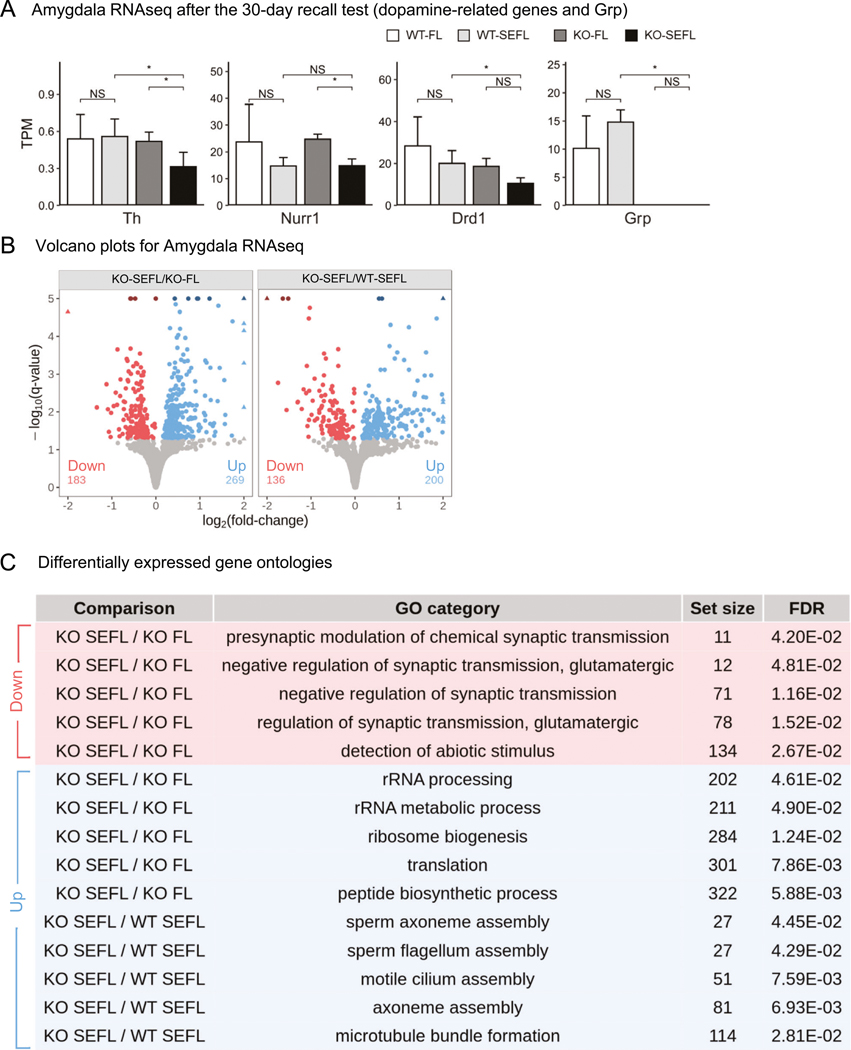
RNA-sequencing of *Grp*^−/−^ mice following recall in SEFL. **A** Expression differences in dopamine signaling-related genes in RNA-seq data recapitulate patterns observed in qPCR datasets. Comparisons of TPM (transcripts per million) based on 4 replicates and (*) indicates significant differences based on *p*-values < 0.05 based on a *t*-test. **B** Results of differential expression for the comparison indicated in the panel headers. Shades of blue and red indicate up and downregulated genes with a *q*-value ≤ 0.05, respectively. To aid in visualization, darker shades indicate points with *q*-values larger than shown and triangular points indicate fold-changes larger than shown, see supplemental data for complete results. The number of up (blue) and downregulated (red) genes is shown at the bottom of each panel. **C** The top 5 GO biological process categories with the smallest set size for each comparison from the list of significant GO categories. The comparison column and color of a row indicate which set of genes were used to find a GO category. Categories with smaller set sizes are shown to increase the specificity of the categories.

To characterize the effects of the *Grp* gene deletion on genome-wide patterns of mRNA expression, we generated RNA-seq datasets following SEFL recall (we used the same time point of SEFL memory recall as in our qPCR analysis above, but we used a separate group of animals) and performed differential gene expression analysis using DESeq2 (Fig. 6). Consistent with our qPCR results, we found that the *Th* has lower abundance (in transcripts per million (TPM)) in the KO-SEFL compared to all the other samples (Fig. 6A) in our RNA-seq datasets. We also found *Nurr1* RNA abundance is lower in SEFL relative to FL in both the wildtype and knockout mice, validating our qPCR results (Fig. 6A). These differences are significant only for the knockout mice likely due to the fact that *Nurr1* abundance in WT-FL group is highly variable. We also find that *Drd1* abundance is significantly lower in KO-SEFL relative to WT-SEFL consistent with our qPCR analysis (Fig. 6A). Some additional dopamine-related genes showed differences in RNA-seq that we did not see in our qPCR analysis: we find that *Drd2* and *Grik2* abundances are significantly lower in KO-SEFL relative to WT-SEFL (Supplementary Fig. 12A). Interestingly, we also find abundance is significantly lower in KO-SEFL relative to WT-SEFL for the *Ppm1f* gene, which encodes protein phosphatase, Mg^2+^/Mn^2+^ dependent 1 F (PPM1F). The *Ppm1f* gene was found to be regulated by stress in mice as well as associated with anxiety and PTSD in humans [53, 59]. While the average abundances of *Ppm1f* in the GRP KO mice across both conditions are lower than in the wildtype control mice, these differences are small and not significant due to high biological variability in mRNA abundances across individual samples in the wildtype mice.

### GRP removal primes dopaminergic system for stress-induced plasticity

We find that in the wildtype mice, SEFL (WT-SEFL) led to no significant changes in gene-expression patterns genome-wide relative to non-stressed fear-conditioned mice that underwent fear extinction (WT-FL; Supplementary Fig. 12B). Similarly, under non-stress conditions, the combination of *Grp* gene deletion and fear conditioning (KO-FL) failed to elicit large changes in gene-expression patterns relative to the wildtype control mice that underwent fear conditioning (WT-FL). However, the combination of stress and fear conditioning (SEFL treatment) drives significant changes in gene-expression patterns in KO-SEFL relative to both WT-SEFL wildtype and KO-FL. Together this indicates that neither the stress alone nor the *Grp* gene deletion by itself has large effects, but that these two conditions can (and perhaps must) act synergistically. More specifically, when comparing KO-SEFL to KO-FL mice (Fig. 6B), we find a modest number of genes with altered expression levels (452, *q*-value < 0.05). Similarly, when comparing KO-SEFL to WT-SEFL mice, we find only 336 genes significantly altered (*q*-value < 0.05) (Fig. 6B). Functional characterization of these differentially expressed genes suggests that genes involved in regulation of synaptic transmission are typically downregulated, while genes involved in microtubule and cilium assembly are upregulated (Fig. 6C). Genes belonging to the sperm assembly categories are microtubule-associated genes with more specific annotations. Overall, these results suggest a specific and consistent functional effect of the *Grp* gene deletion on synaptic regulation and microtubule-associated genes. Furthermore, many of the genes that were significantly affected were associated with dopamine signaling and recapitulated effects seen in our behavioral and qPCR analyses. Taken together, these results suggest that the GRP may link stress to fear through the modulation of the dopamine signaling.

## DISCUSSION

### The GRP regulates dopamine signaling in opposite directions in fear learning and extinction

We have identified and characterized the GRP as a molecular link regulating dopaminergic control of fear learning and extinction. When this molecular link is disrupted, dopamine is enhanced in the amygdala when the tone-CS or shock-US are presented during fear learning and when tone-CS is presented during extinction on day 1, while dopamine-related gene transcription is decreased following CS memory recall of fear extinction (Supplementary Fig. 13). Combined optogenetics and ex vivo electrophysiology suggest disrupted VTA-BLA connectivity in *Grp*^−/−^ mice, demonstrating an importance of the GRP for the input from the VTA to BLA. The dependence of BLA function on the VTA inputs as well as changes in dopamine signaling at the early and late stages of fear learning and extinction provide an initial insight into how the GRP may regulate dopamine function and confer susceptibility to exaggerated fear in animals and possibly PTSD in humans. Also, our study confirms recently emerging work, showing that the GRP links stress and fear processing [60, 61].

### The GRP may serve as a functional biomarker of learned fear processing

Several studies show the importance of dopamine neurons in the VTA during fear extinction [22, 24]. Importantly, the *Grp* mRNA is a selective marker for dopaminergic subpopulations in the VTA both in mice and humans [39, 62, 63]. Moreover, our results support recent work that dopamine may play a more general role in salience signaling [64] and suggest that the GRP-dopamine link is important for the initial encoding of fear memory as well as for the long-term memory recall in fear extinction processes. Thus, if deficiencies in GRP support aberrant dopaminergic signaling and enhanced salience at the time a fear memory is acquired, it may serve as an important marker for the long-term susceptibility to protracted fear responses and mental illness.

Within the amygdala-associated neural circuitry, the GRP is released from excitatory neurons and binds to the GRP receptor expressed exclusively by GABAergic interneurons stimulating GABA release and inhibiting principal neurons [38, 45, 65–67]. The lack of the GRPR leads to an increase in long-term potentiation in the BLA, enhanced fear memory and deficits in fear extinction [38, 45]. However, within the VTA, less is known about the functional role of the GRP, besides its almost exclusive co-localization with markers of dopaminergic neurons [39, 40]. Nonetheless, one interesting possibility given the role of GRP in the BLA and the observed increase in excitatory potentials that we observed here is that GRP could be transmitted by VTA neurons that co-transmit dopamine and glutamate [63, 68], some of which also participate in salience signaling [69].

In addition to assessing the GRP-dopamine link, the *Grp*^*−/−*^ mice allowed for mapping the learned fear circuitry of the tone-CS-specific projections entering the amygdala from the auditory thalamus and auditory cortex. Combining visualization of the GRP-positive cells by the GFP knocked-in into the *Grp* gene locus in the *Grp*^*−/−*^ mice together with rAAV2-retro-based tracing [46], we were able to examine which of these two auditory pathways contain the GRP. The results demonstrate that the GRP labels only one of the two pathways from the auditory thalamus to the amygdala:the GRP labels the multisynaptic indirect thalamo-cortico-amygdala pathway but not the direct thalamo-amygdala pathway [3, 47]. Overall, because the GRP is selectively present in the neural circuitry regulating fear learning and extinction – the BLA, auditory thalamus, auditory cortex, VTA, ventral hippocampus and mPFC [38, 45, 70, 71] – it is a unique functional biomarker of learned fear processing.

### The *Grp*^−/−^ mice may serve as a genetic model of PTSD-like symptoms

Our data suggest that the *Grp*^−/−^ mice can be used as a genetic model of PTSD symptoms. Naïve *Grp*^−/−^ mice displayed normal anxiety and pain sensitivity (Supplementary Fig. 3 and 5), consistent with normal expression of immediate-early genes (IEG; *c-Fos* and *Arc*; Supplementary Fig. 4) and dopamine-related genes (*Drd1*, *Drd2*, *Th* and *Nurr1*; Supplementary Fig. 9) in the BLA and VTA of naïve *Grp*^−/−^ mice. These behavioral and gene expression data suggest that delayed extinction observed in SEFL in *Grp*^−/−^ mice is likely to reflect changes in stress-enhanced learning and memory processes rather than in anxiety in naïve state. It appears that both learning and extinction of fear are affected by the GRP-dopamine interactions. Importantly, fear extinction is strongly dependent on initial fear learning – so, it is expected to see an effect in both processes, when a manipulation is done before fear testing (fear learning and fear extinction) is conducted (unless there is a specific experimental design that affects only extinction, for example). Moreover, examining individuals with different levels of initial fear learning and their subsequent differences in extinction is also highly translationally relevant, as these are precisely the differences that recapitulate pathological fear responses. Overall, our transgenic mouse model supports the idea that preexisting changes in genes and neural circuits may predict and cause deficits in PTSD in humans [72, 73].

Delayed extinction in the *Grp*^−/−^ mice appears to be long term, as evident in the enhanced freezing during the recall test that is performed three-four weeks following the initial stages of SEFL: acute stress, fear learning and extinction. Our current data provide genetic evidence that a decrease in the GRP signaling leads to deficiency in fear extinction, whereas an increase in the GRP signaling improves fear extinction [38, 45, 74–77]. While the SEFL paradigm clearly showed fear learning and extinction deficits in the *Grp*^*−/−*^ mice vs. their wildtype counterparts as well as it showed deficits in the susceptible group compared to the resilient group of wildtype mice over two days of fear extinction and during the Recall session (Fig. 2H), we did not find that stress induces fear extinction deficits in wildtype mice as a whole group, as we reported earlier [52]. It is possible that because we kept both the knockout and wildtype mice together, these two groups influenced each other, as mixed-group housing of knockout and wildtype mice was reported previously to affect behavior [78].

Recent work provides clear evidence of the importance of the VTA in fear extinction, with the VTA sending dopamine projections to other brain areas critically involved in fear extinction [79, 80]. While recent work has focused on the dopamine-related neural circuitry of fear extinction, we know very little about the molecular pathways supporting the role of dopamine in this process. Our work here begins to address this, by describing how the GRP may regulate dopamine function in the VTA-BLA circuitry. Combined optogenetics and ex vivo electrophysiology show that in the absence of the GRP, the BLA is overexcited, and the VTA-BLA connectivity is disrupted and cannot be activated under conditions we tested. Altogether these results support the notion that the GRP may have a specific role in modulating the dopamine control of fear extinction.

### Dopamine release is enhanced during fear learning and early phases of extinction, but dopamine-related gene transcription is down following extinction memory recall in the *Grp*^−/−^ mice

The increased release of dopamine observed during shock presentation during initial fear conditioning and extinction on day 1 suggests that excessive release of dopamine may have led to a down regulation of dopamine-related genes to compensate for increased availability of dopamine in the *Grp*^*−/−*^ mice. Compensatory changes in gene expression of dopamine receptors have been reported when dopamine availability is altered [81, 82]; however, more research is needed to explore this mechanism in the VTA-BLA circuit of the *Grp*^*−/−*^ mice. Intriguingly, dopamine-related gene transcription was normal in naïve *Grp*^*−/−*^ mice, but following Recall, gene transcription of *Th*, *Nurr1*, *Drd1* and *Drd2* is decreased. While more experiments are required to better understand when and how the lack of the GRP may modify BLA synaptic activity and gene expression, our results demonstrate the importance of stress and fear experiences in these processes.

A decrease in the *Th* mRNA levels in the BLA of the *Grp*^*−/−*^ mice following SEFL recall suggests that the *Th* mRNA, a critical enzyme involved in dopamine synthesis, was located in synapses of the VTA presynaptic neurons projecting to the BLA, confirming previous work [54, 55, 83]. In support of our finding of the *Drd1* mRNA, previous work showed that the D1 receptors expressed in the BLA [21, 23, 32, 34, 84–87]. Another gene we found codes for the NURR1, a transcription factor and an IEG located in the cell nucleus; thus, the changes we see in the NURR1 are in the BLA neurons. NURR1 is known to modulate dopamine signaling [88–92]. Overall, these RNA transcription data suggest that GRP’s influence on dopamine may influence the acquisition and extinction of stress-enhanced fear memories and indicate for the first time that therapies targeting GRP as a potential dopamine-oriented drug in psychotherapy approaches to maximizing consolidation of successful fear extinction and safety learning [93]. Interestingly, an additional support for the idea that the *Grp*^−/−^ mice may be a genetic model for PTSD is provided by the fact that we found the *Ppm1f* gene to be significantly decreased in the *Grp*^−/−^ mice following SEFL. The *Ppm1f* gene is regulated by stress in mice and is associated with anxiety, depression and PTSD in humans [53, 59].

A limitation in the interpretation of our data is that the *Grp*^*−/−*^ mice are generated using the classical gene knockout method, which means the gene is removed from every cell of the body and during early embryo development. To mitigate some of these concerns, we used the toxin-based removal of the GRPR-positive interneurons in the amygdala of the adult mice. While we were able to replicate the GRP removal effects on fear conditioning, this doesn’t fully address this concern. Thus, future works is required to better understand where and when the loss of GRP/GRPR leads to changes in the VTA-BLA synaptic connectivity and gene expression in the amygdala, and how to link these changes to the behavior.

It is important to emphasize that the molecular and behavioral changes we observe in the *Grp*^*−/−*^ mice suggest that the GRP may link stress and fear. An acute restraint stress elicits the release of both corticotropin-releasing factor (CRF) and GRP in the amygdala [94]. The *glucocorticoid receptor* gene knockout in the GRPergic neural circuitry by Inoue et al. [60] and work by Goto et al. [61] also demonstrate that the GRP is critically involved in integrating stress and fear processing [95]. Since our paper focuses on the GRP-dopamine connection, we did not study other neurotransmitters in the *Grp*^*−/−*^ mice. The GRP seems to regulate other molecular pathways as well, as seen in our RNA-seq data (Fig. 6B, C). Also, our electrophysiological studies suggest that glutamatergic synaptic circuits are affected downstream of the VTA-BLA projections in the *Grp*^*−/−*^ mice and can add to the effects described here. Overall, our results suggest that enhancing dopamine function long-term by increasing transcription of dopamine-related genes can improve extinction of undesired fear, which may have clinical relevance for PTSD. Therefore, GRP- and dopamine-transcription-based approaches can be used together with exposure-based cognitive behavior therapy in extinction-impaired individuals.

## Supplementary Material

RNAseq related files (Suppl Tables 1, 2 and 3)

2

## Data Availability

All the data used in this study are included within the manuscript’s figures or provided in the supplementary information section and Source Data files. The raw sequencing data are deposited under the GEO accession GSE164226. Any additional data and information are available upon request to the corresponding authors, Drs. David Barker, Premal Shah, Juan Marcos Alarcon and Gleb P. Shumyatsky. Source data are provided with this paper.
