# Supplementary material for: Dopamine release and dopamine-related gene expression in the amygdala are modulated by the gastrin-releasing peptide in opposite directions during stress-enhanced fear learning and extinction
Source: Mol Psychiatry. 2024 Nov 23;30(6):2381–94. doi: 10.1038/s41380-024-02843-8 (PMC12092189; doi:10.1038/s41380-024-02843-8)
Supplement: Supplementary file 1 — Supplementary Figures [file 41380_2024_2843_MOESM1_ESM.pptx]

## Slide 1
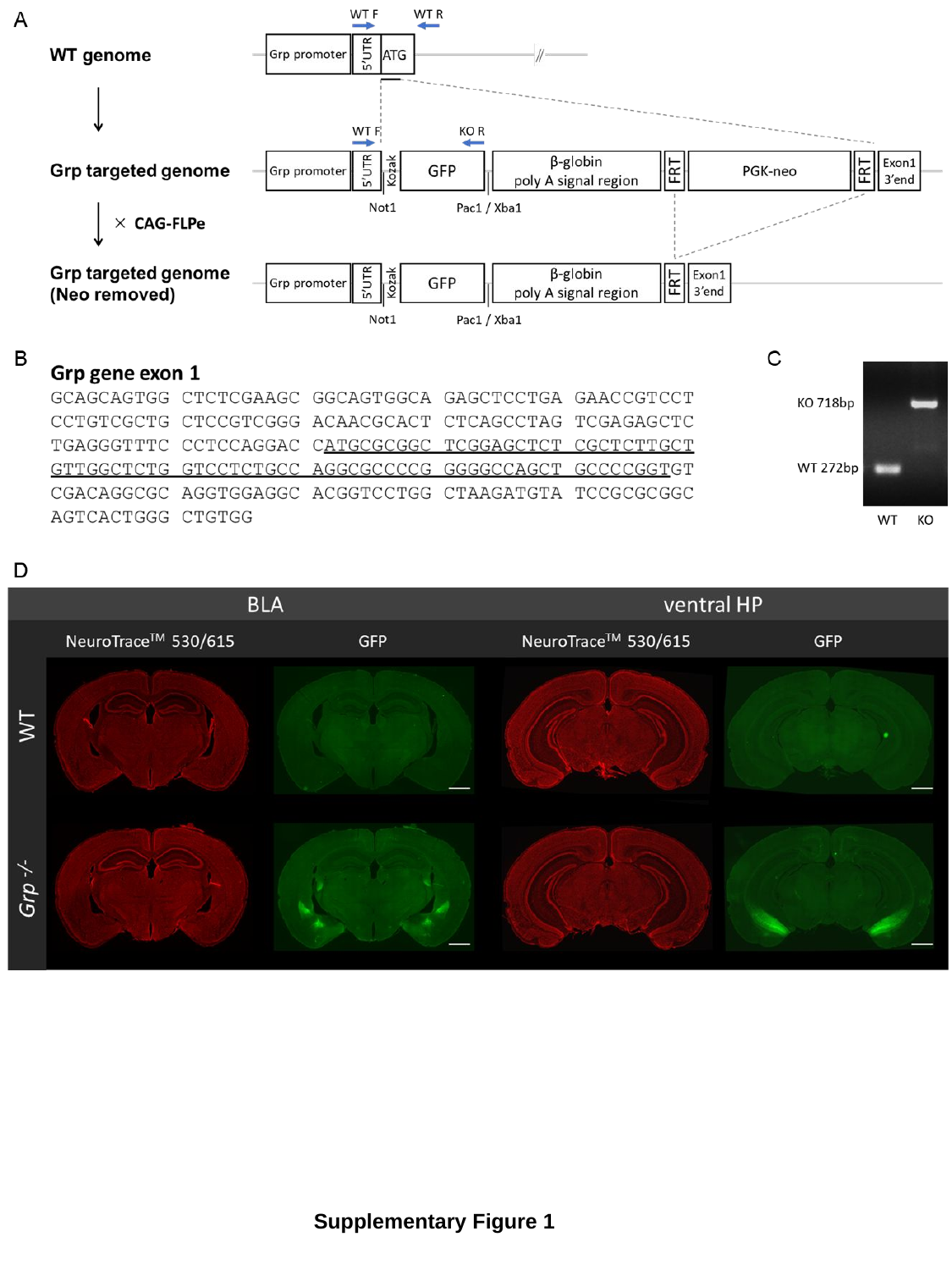

Supplementary Figure 1

## Slide 2
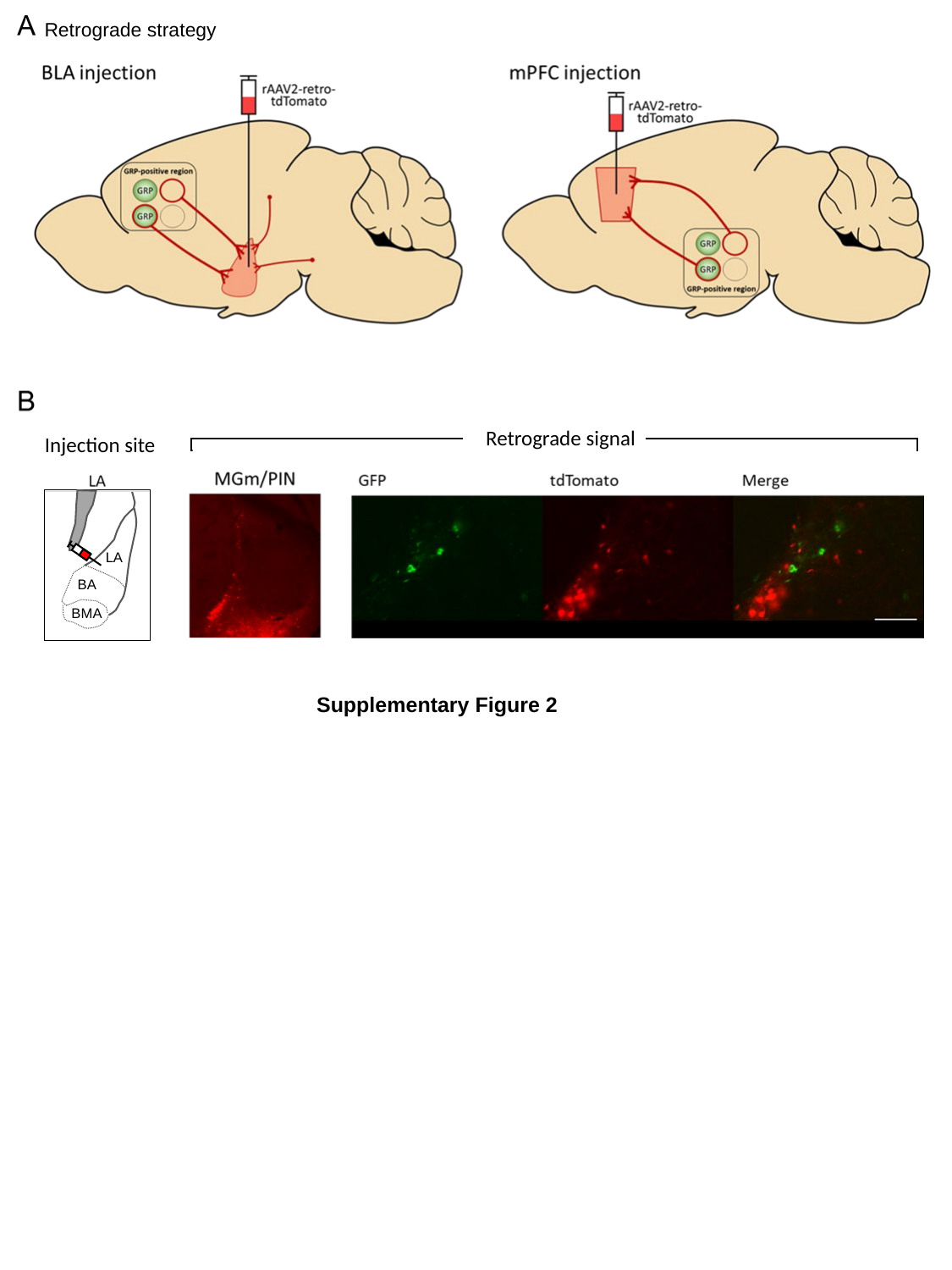

Retrograde strategy
Retrograde signal
Injection site
LA
BA
BMA
Supplementary Figure 2

## Slide 3
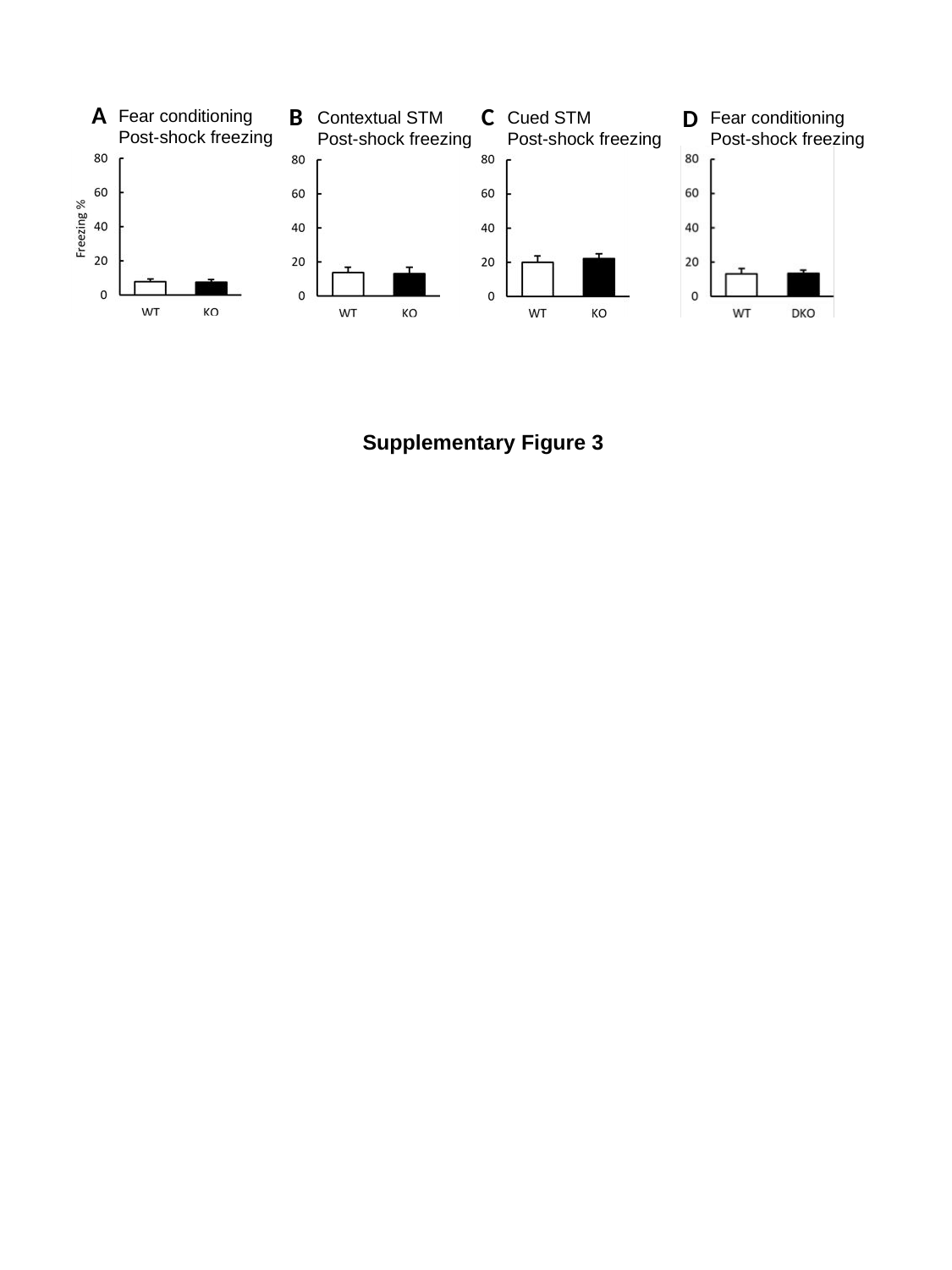

A
B
C
D
Fear conditioning
Post-shock freezing
Contextual STM
Post-shock freezing
Cued STM
Post-shock freezing
Fear conditioning
Post-shock freezing
Supplementary Figure 3

## Slide 4
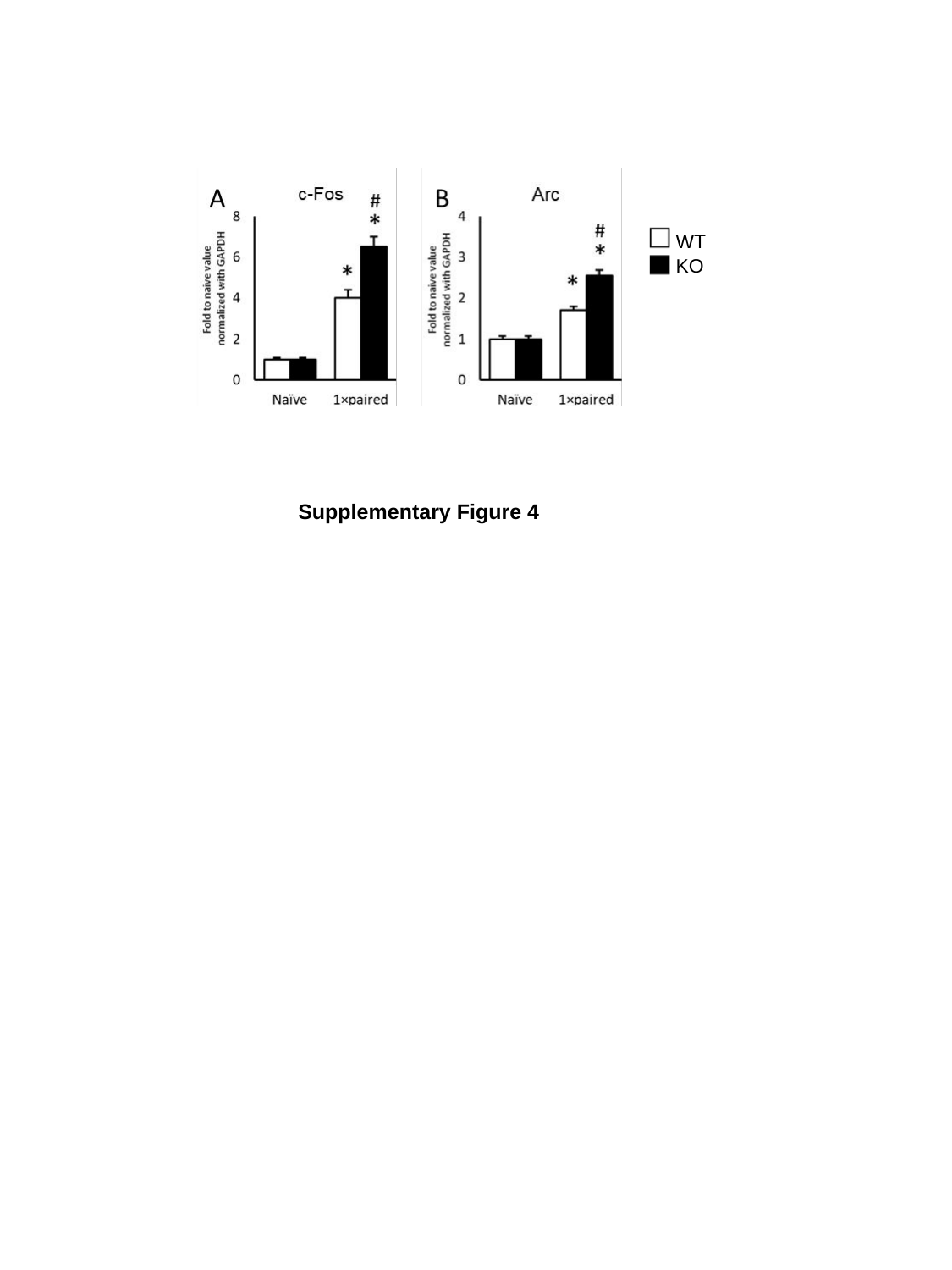

Supplementary Figure 4
WT
KO

## Slide 5
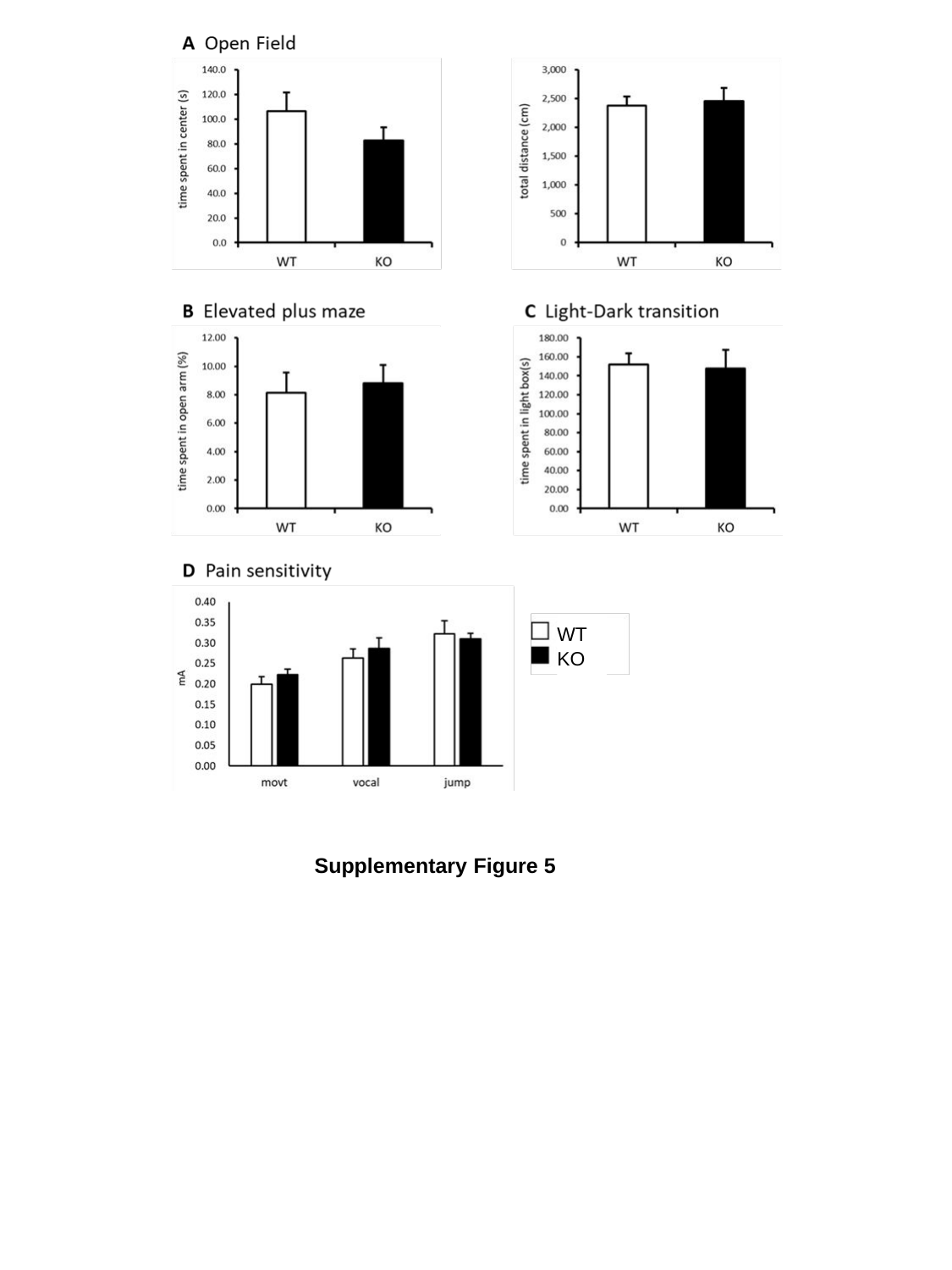

Supplementary Figure 5
WT
KO

## Slide 6
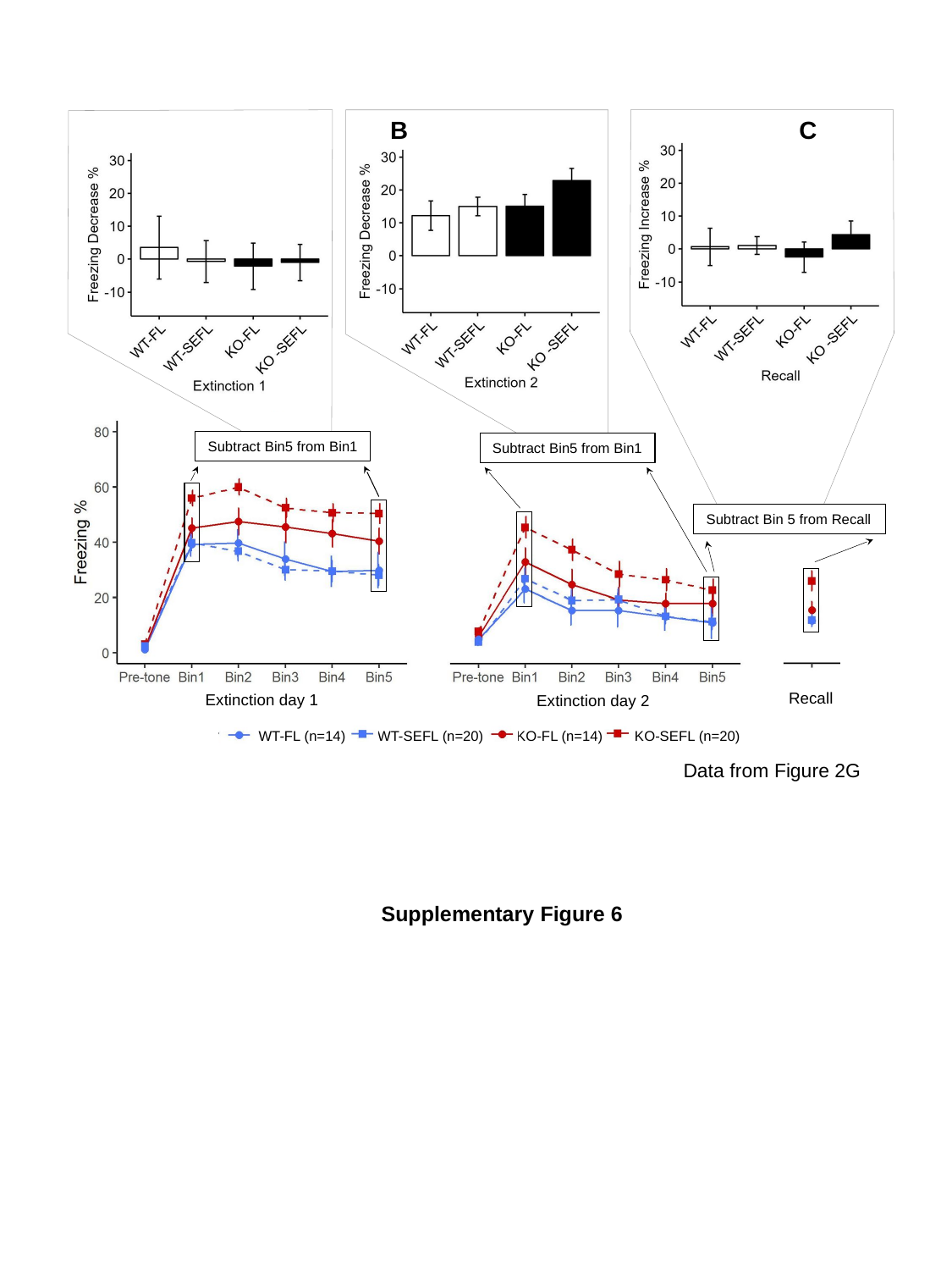

A			 B			 C
Subtract Bin5 from Bin1
Subtract Bin5 from Bin1
Subtract Bin 5 from Recall
Recall
Extinction day 1
Extinction day 2
WT-FL (n=14) WT-SEFL (n=20)	 KO-FL (n=14) KO-SEFL (n=20)
Data from Figure 2G
Supplementary Figure 6

## Slide 7
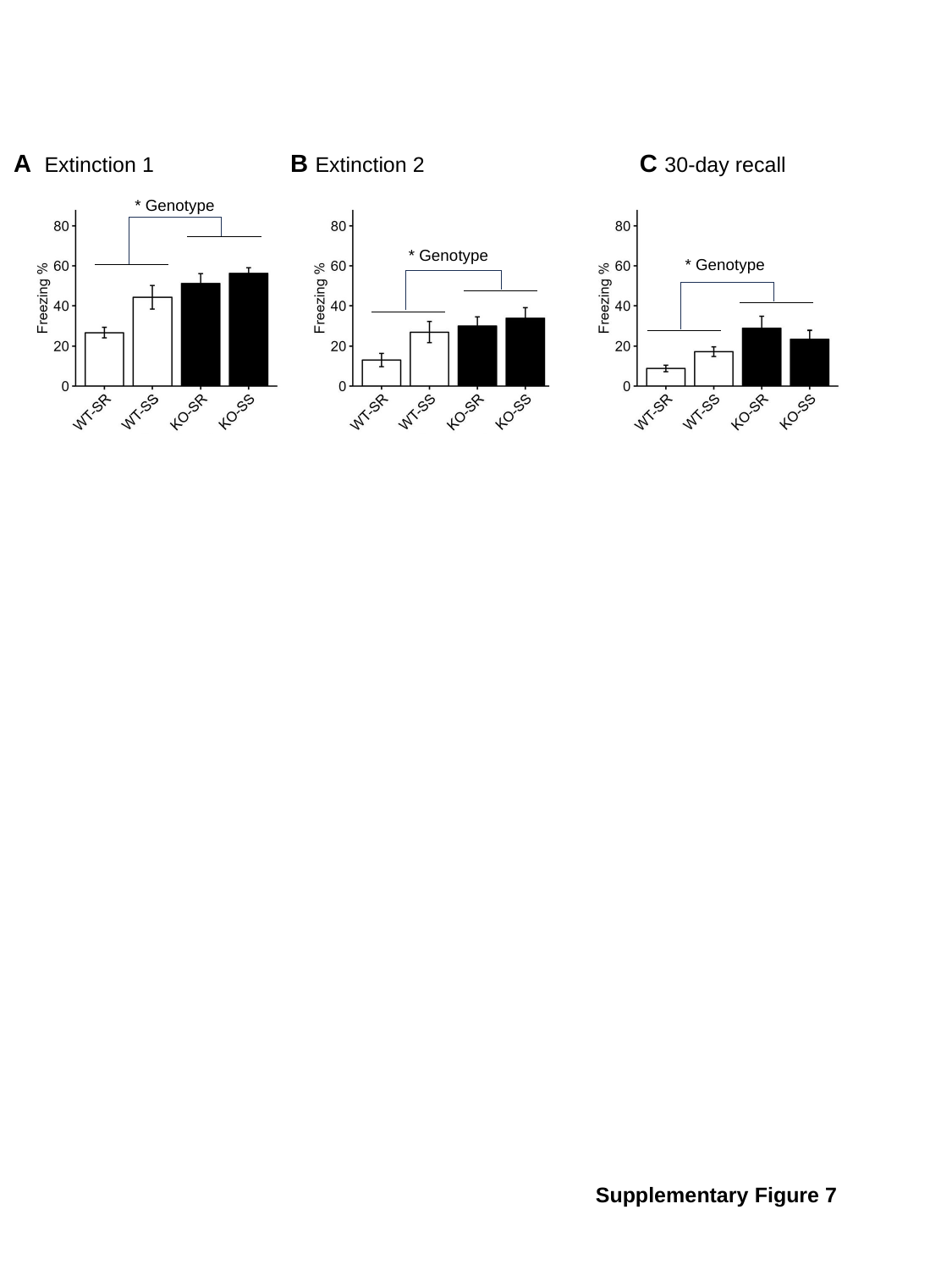

A Extinction 1 B Extinction 2	 C 30-day recall
* Genotype
* Genotype
* Genotype
Supplementary Figure 7

## Slide 8
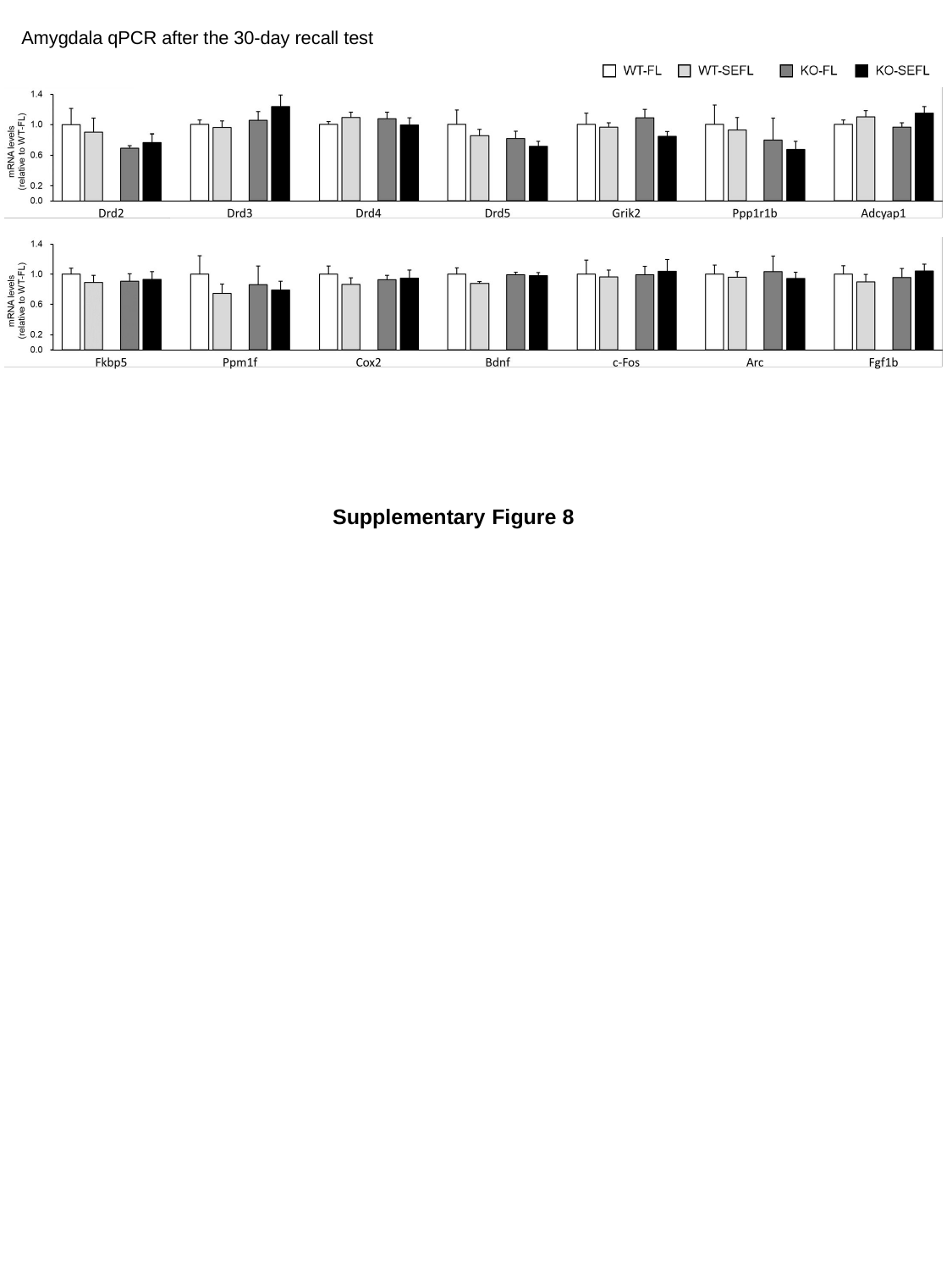

Amygdala qPCR after the 30-day recall test
Supplementary Figure 8

## Slide 9
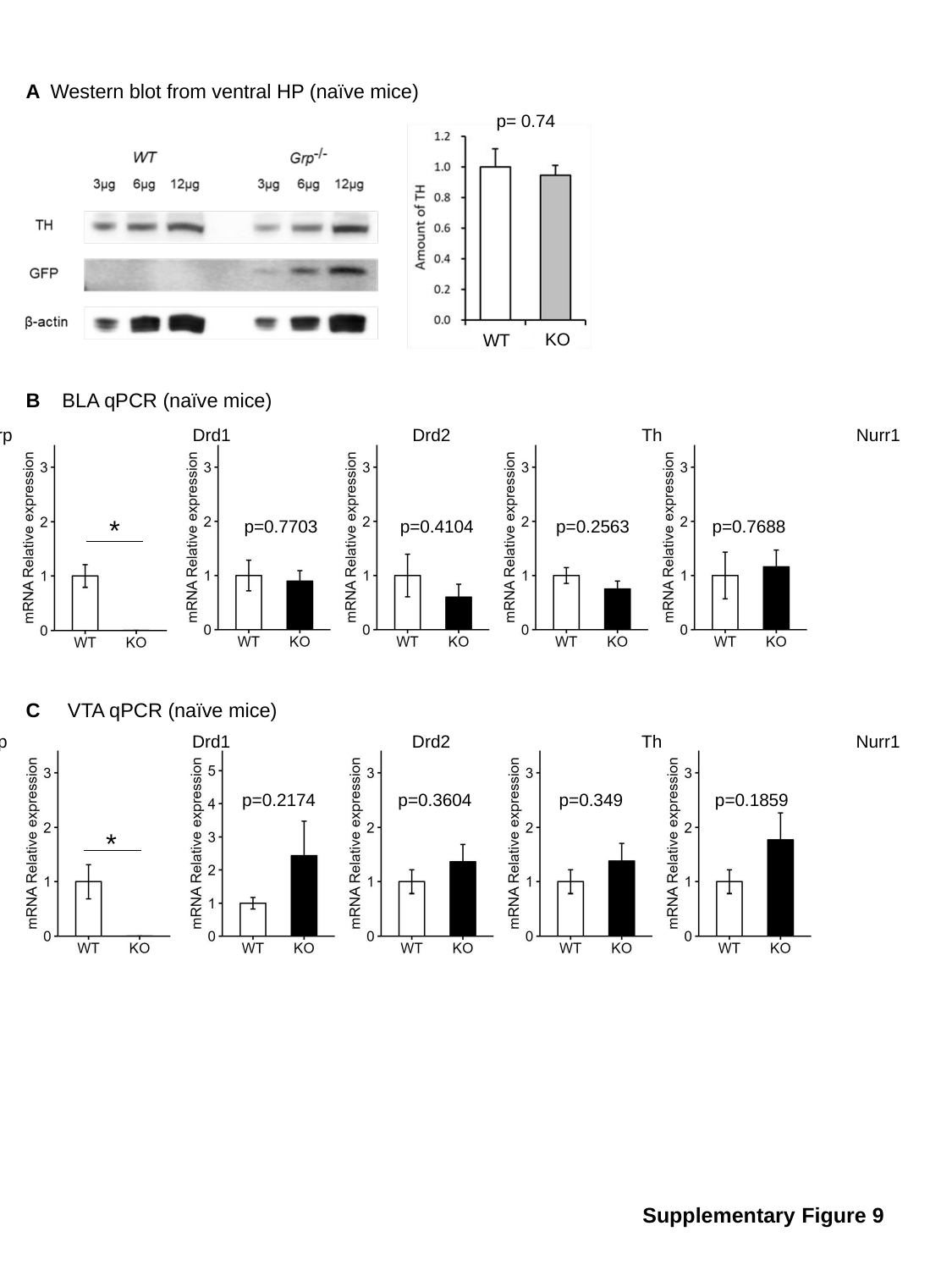

A Western blot from ventral HP (naïve mice)
B BLA qPCR (naïve mice)
C VTA qPCR (naïve mice)
p= 0.74
 KO
WT
Grp	 Drd1		 Drd2		 Th	 Nurr1
*
p=0.7703 p=0.4104 p=0.2563 p=0.7688
Grp	 Drd1		 Drd2		 Th	 Nurr1
p=0.2174 p=0.3604 p=0.349 p=0.1859
*
Supplementary Figure 9

## Slide 10
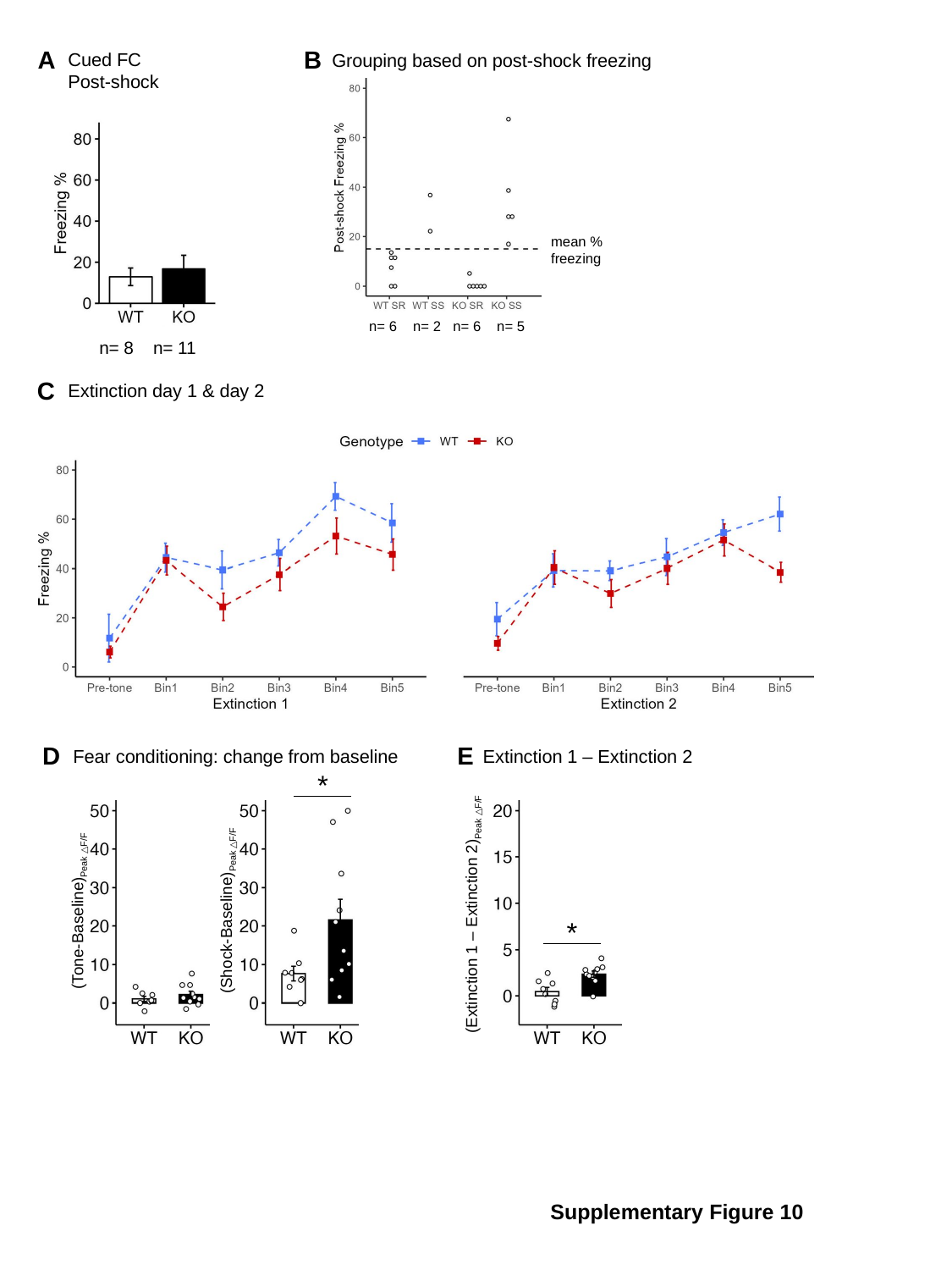

A
B
Cued FC
Post-shock
Grouping based on post-shock freezing
mean %
freezing
n= 6 n= 5
n= 6 n= 2
n= 8 n= 11
C
Extinction day 1 & day 2
D E
Fear conditioning: change from baseline
Extinction 1 – Extinction 2
*
 (Tone-Baseline)Peak △F/F
 (Shock-Baseline)Peak △F/F
(Extinction 1 – Extinction 2)Peak △F/F
*
Supplementary Figure 10

## Slide 11
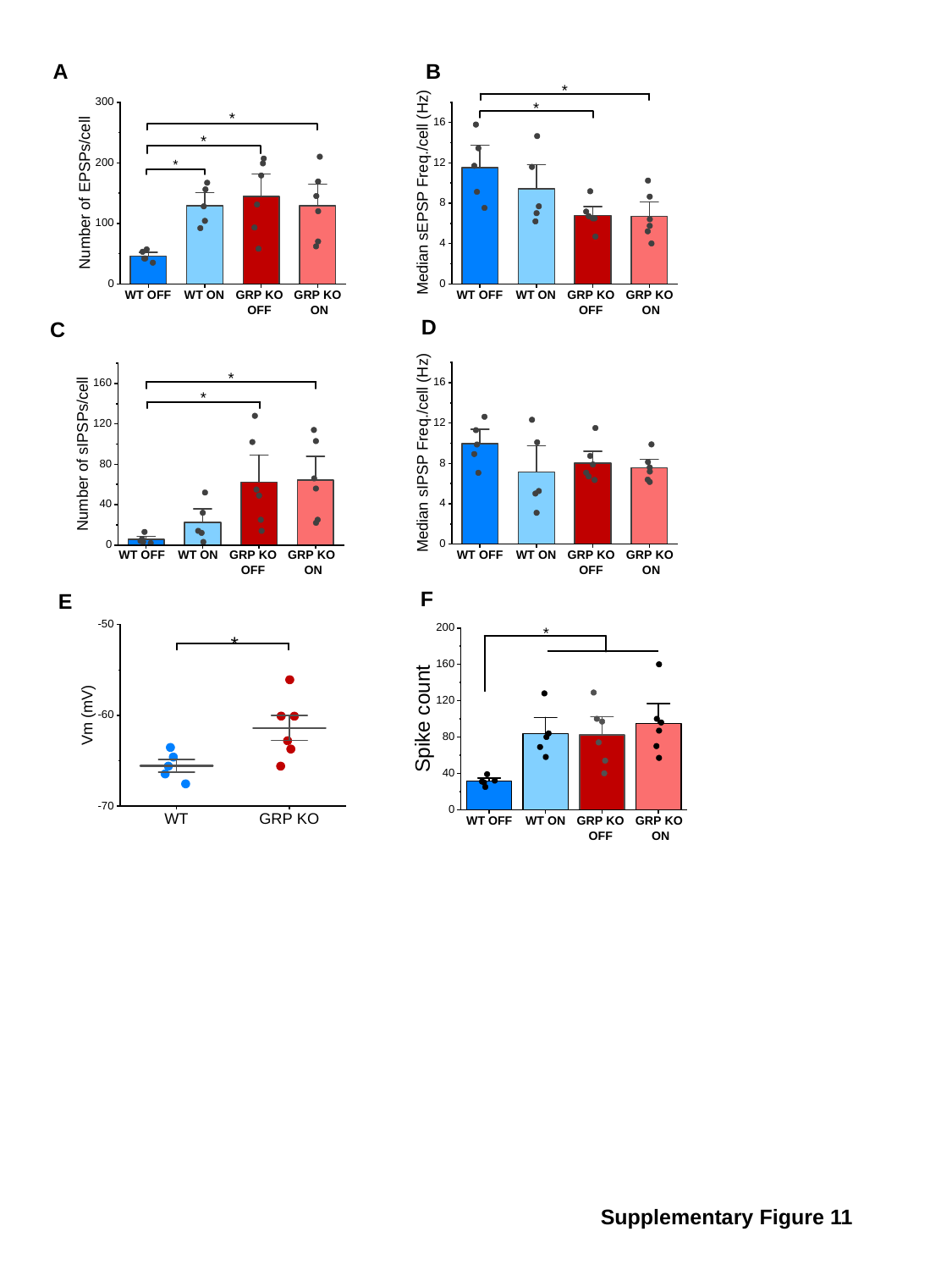

A
B
D
C
F
E
Supplementary Figure 11

## Slide 12
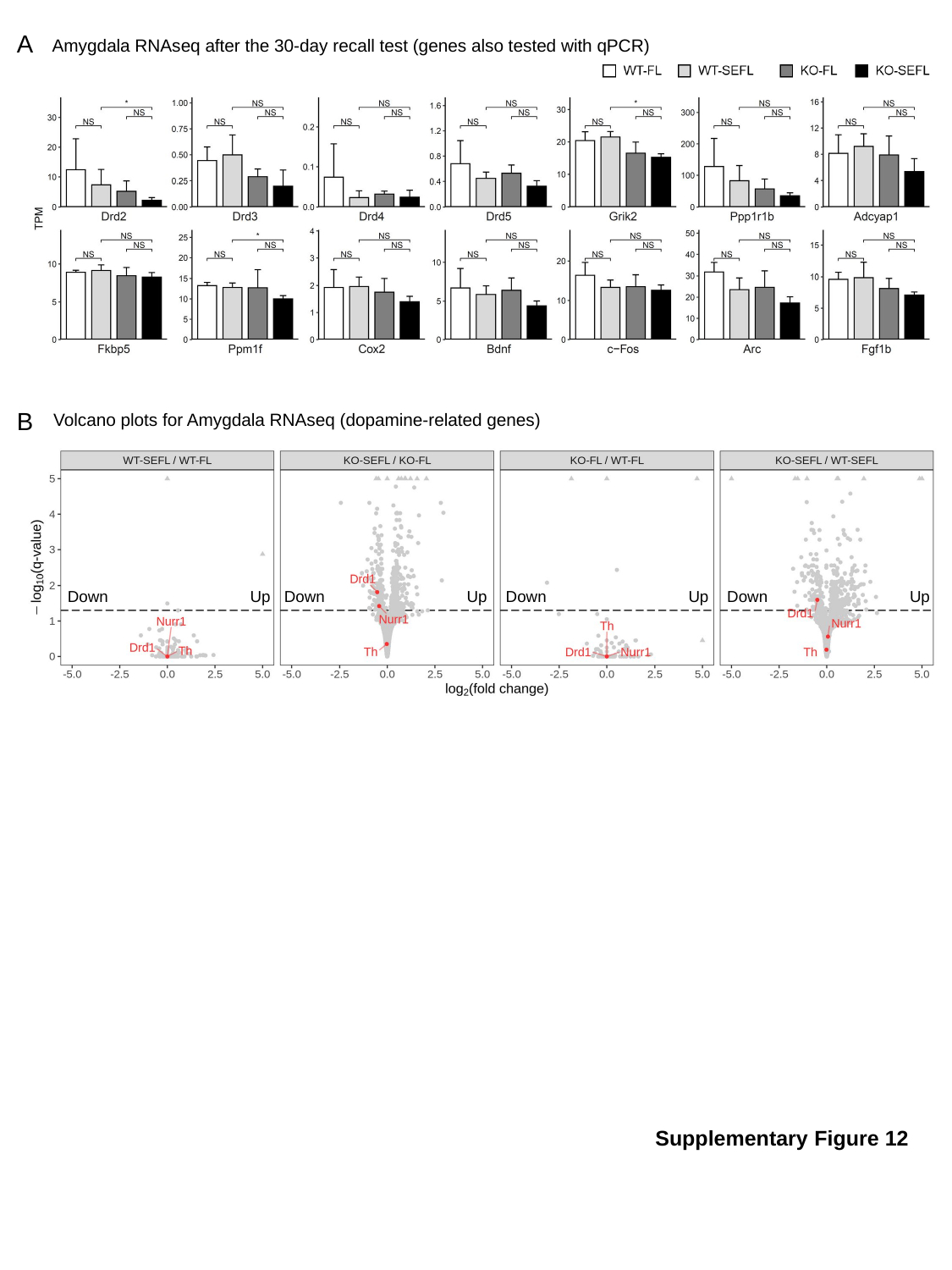

A
B
Amygdala RNAseq after the 30-day recall test (genes also tested with qPCR)
Volcano plots for Amygdala RNAseq (dopamine-related genes)
Down Up
Down Up
Down Up
Down Up
Supplementary Figure 12

## Slide 13
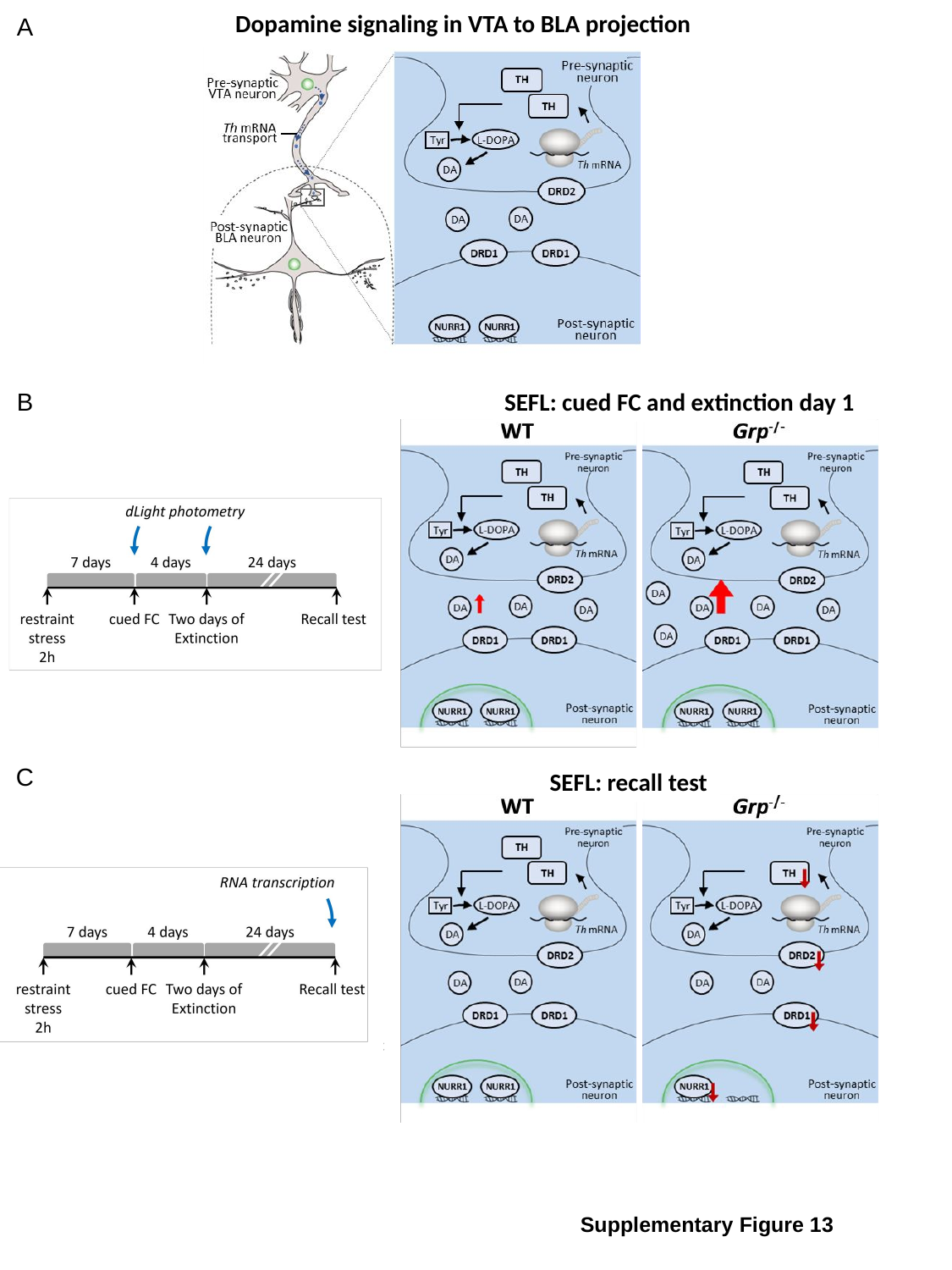

Dopamine signaling in VTA to BLA projection
A
B
SEFL: cued FC and extinction day 1
C
SEFL: recall test
Supplementary Figure 13

## Slide 14
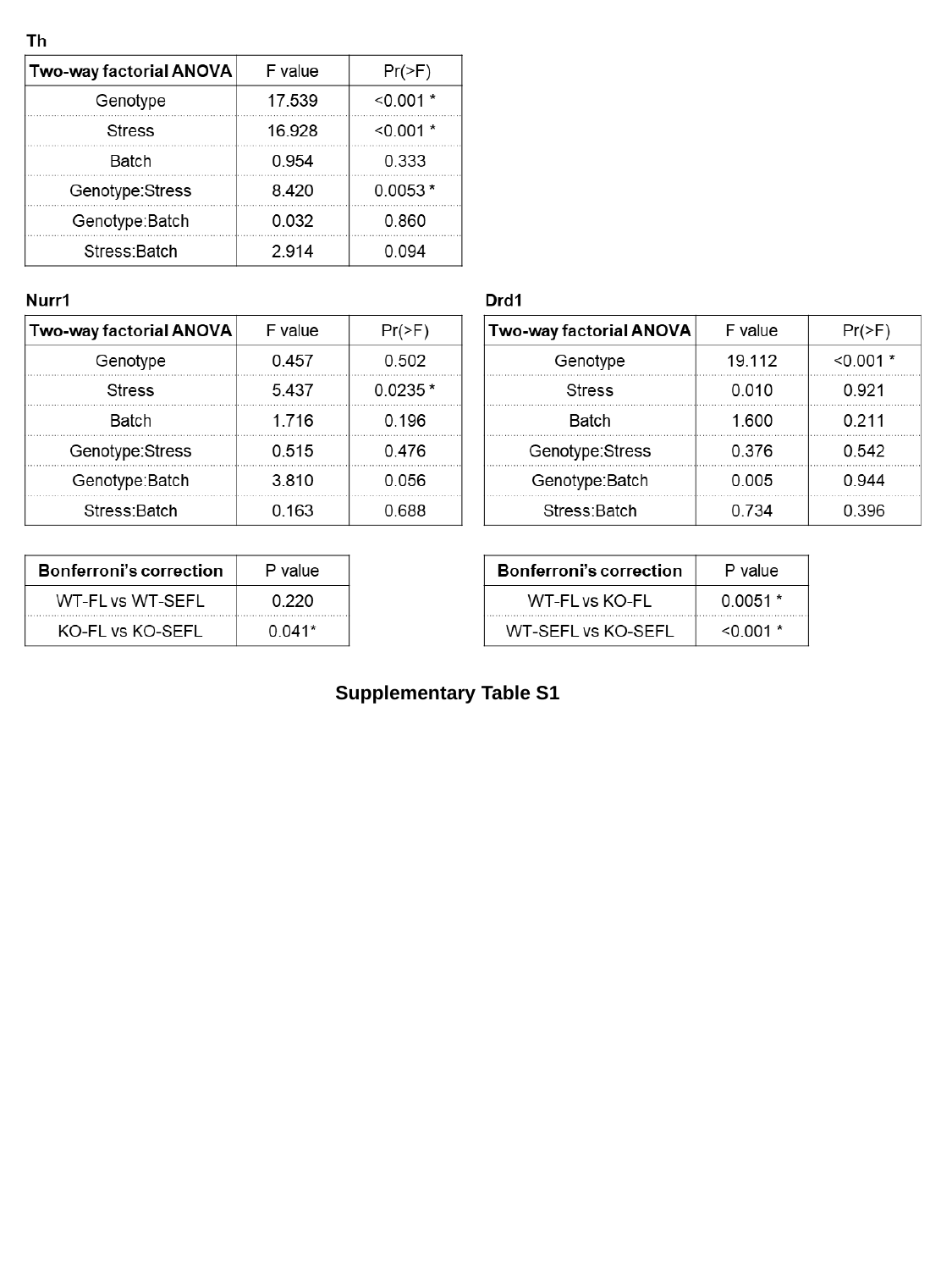

Supplementary Table S1
