# Supplementary material for: Dopamine release and dopamine-related gene expression in the amygdala are modulated by the gastrin-releasing peptide in opposite directions during stress-enhanced fear learning and extinction
Source: Mol Psychiatry. Author manuscript; Available in PMC 2025 Jun 1. (PMC12092189; doi:10.1038/s41380-024-02843-8)
Supplement: Supplementary file 1 [file NIHMS2066436-supplement-2.pdf]

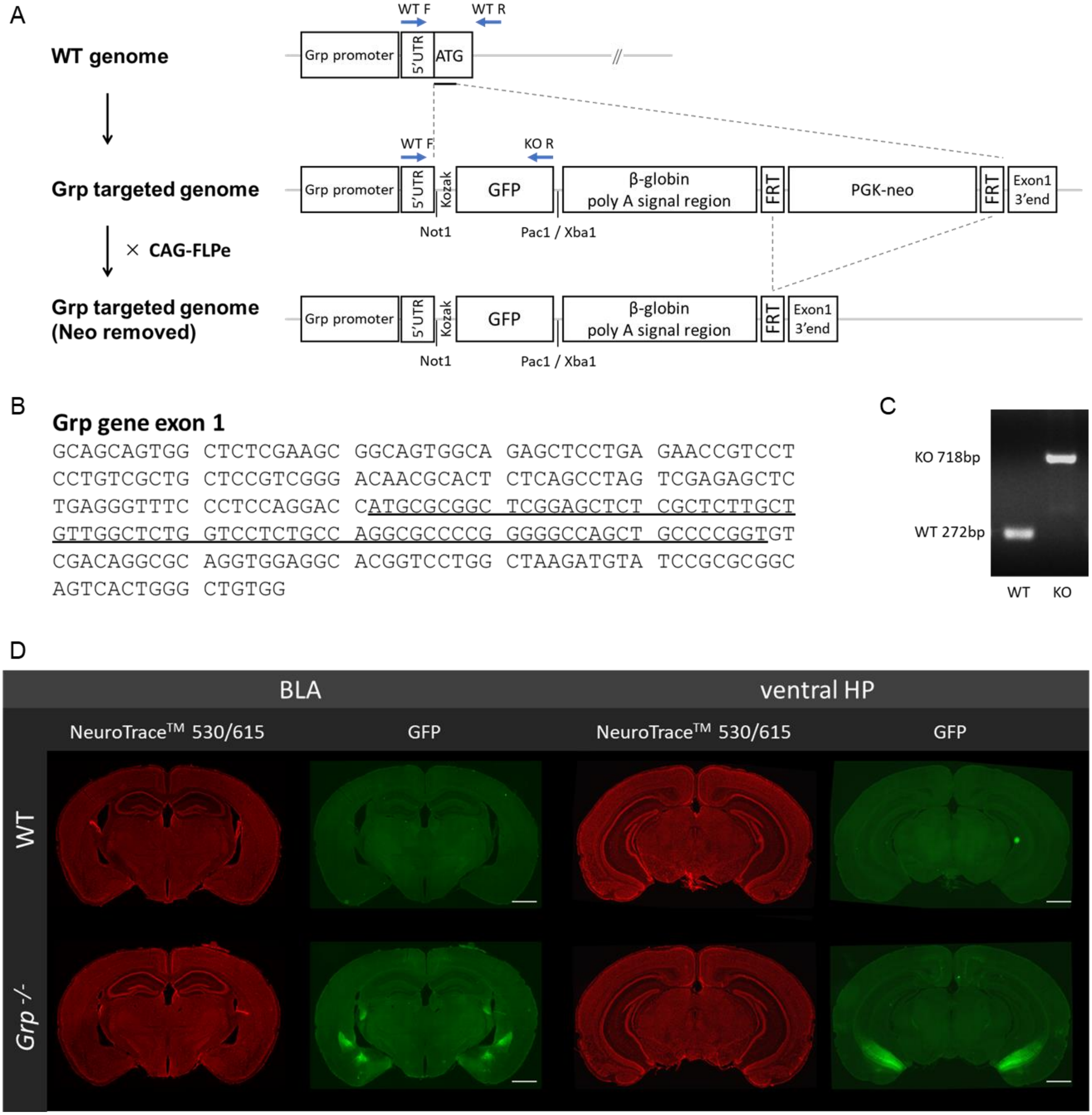

Supplementary Figure 1

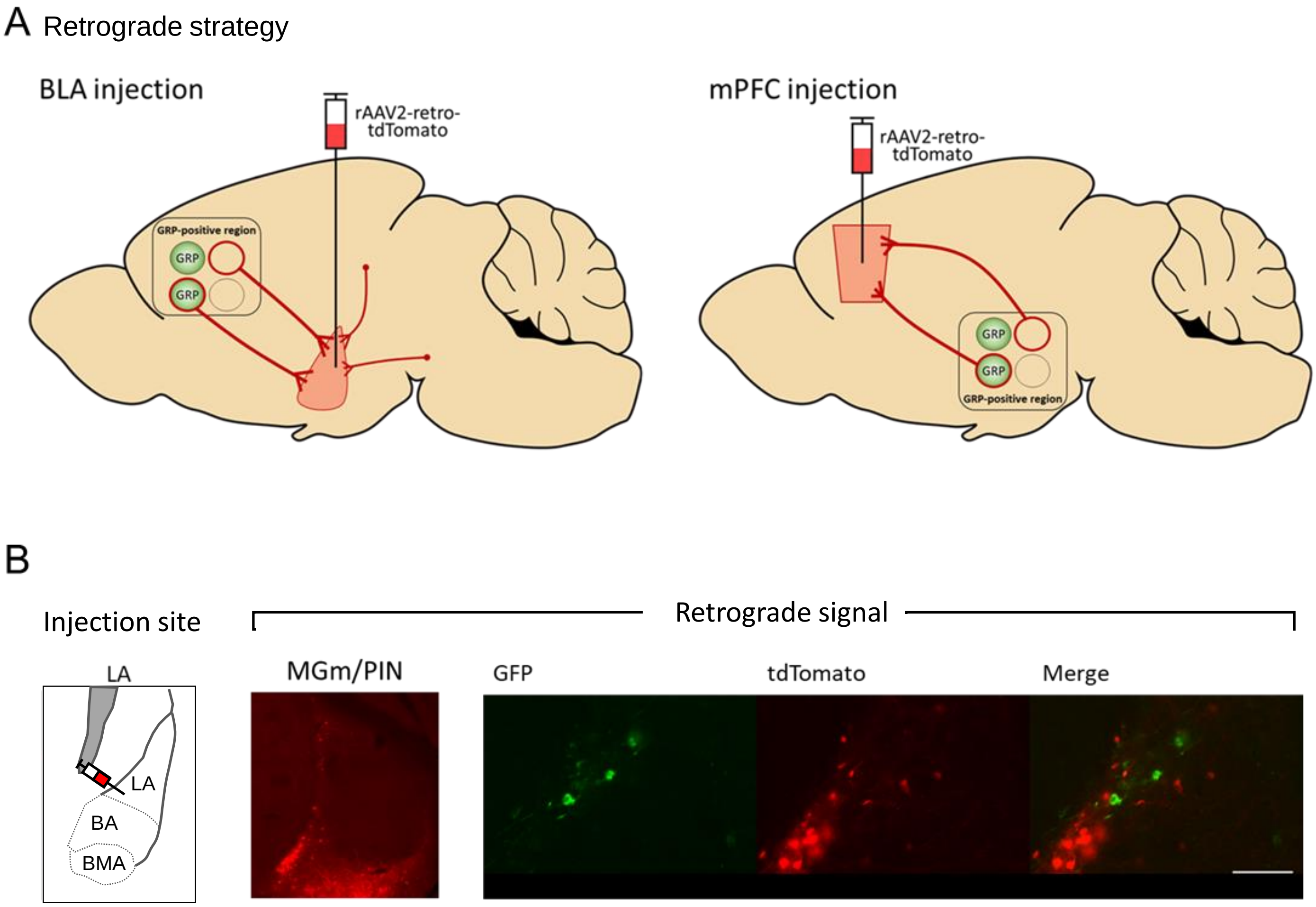

Supplementary Figure 2

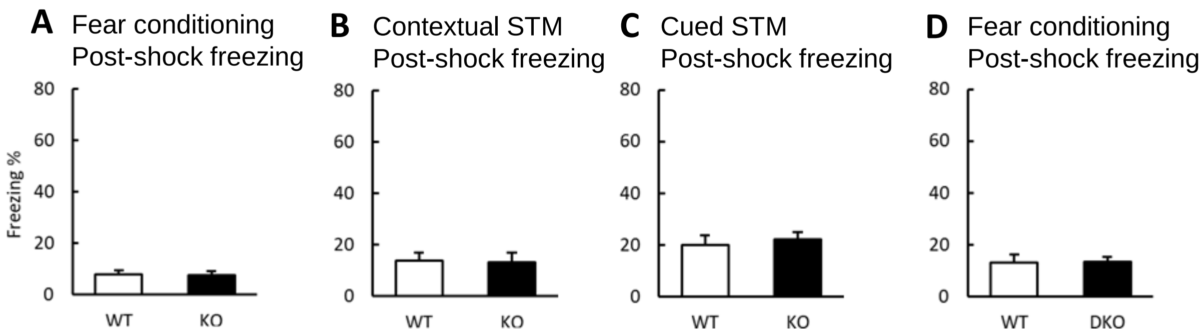

Supplementary Figure 3

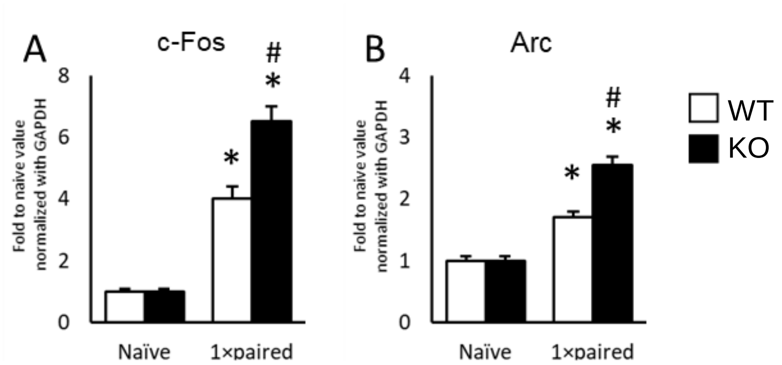

Supplementary Figure 4

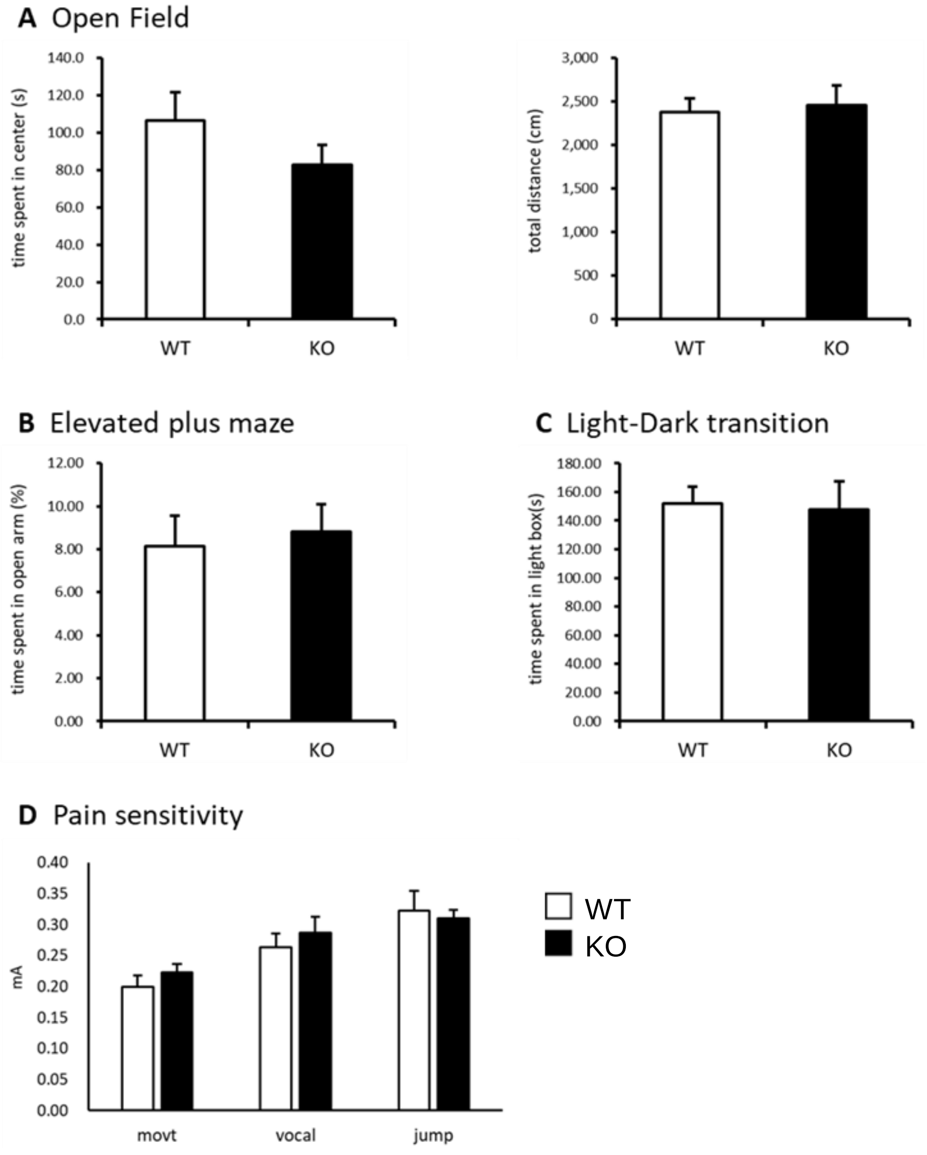

Supplementary Figure 5

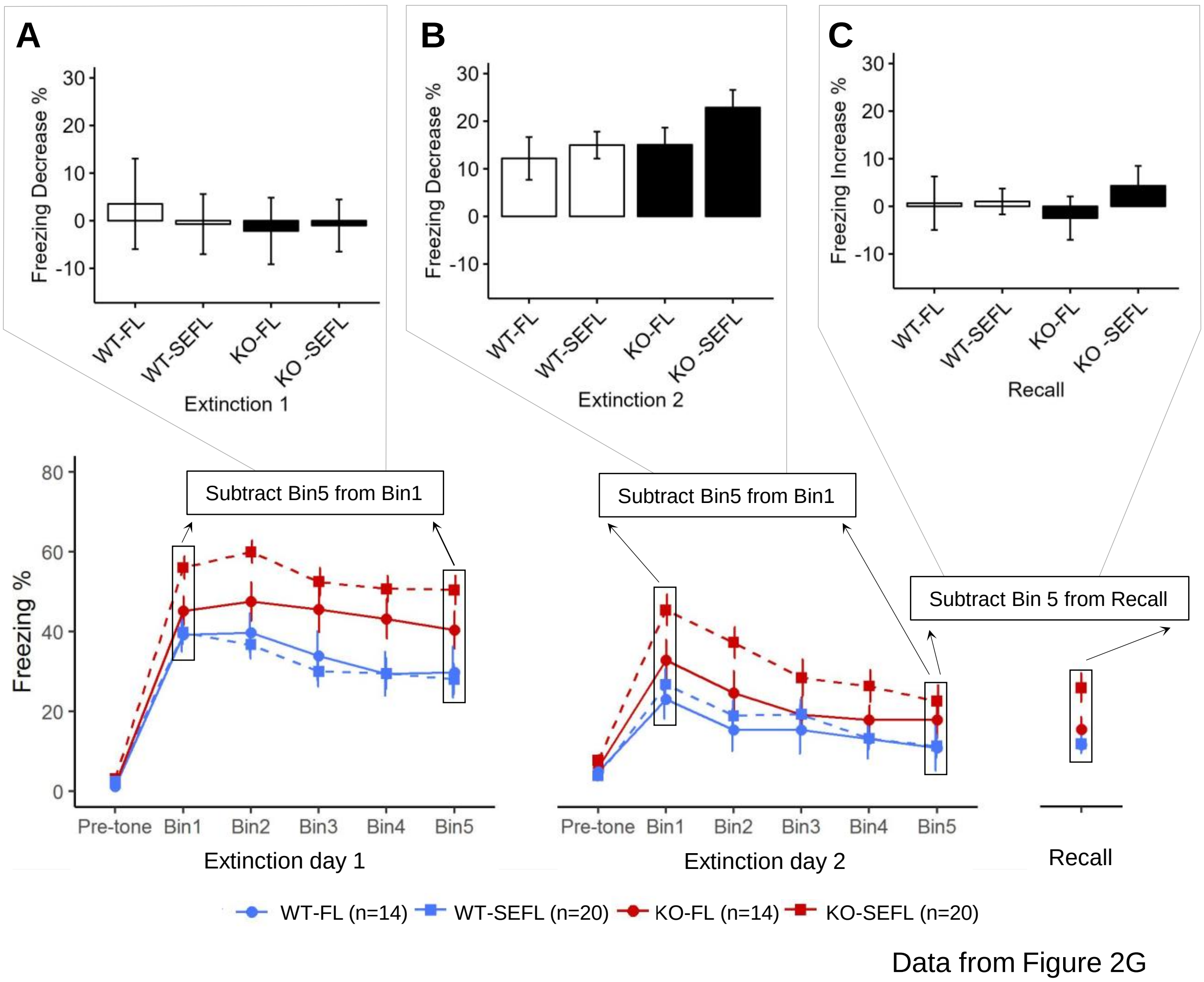

Supplementary Figure 6

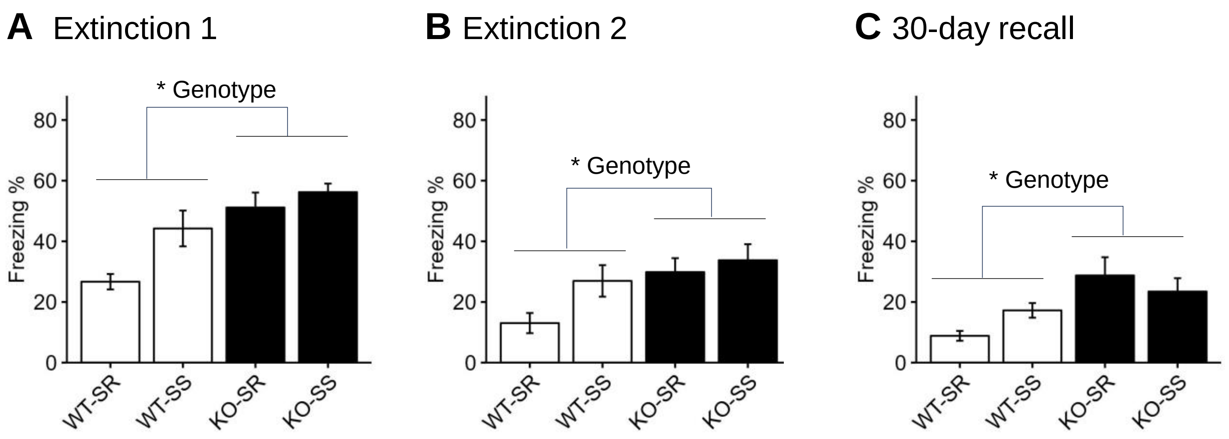

Supplementary Figure 7

Amygdala qPCR after the 30-day recall test

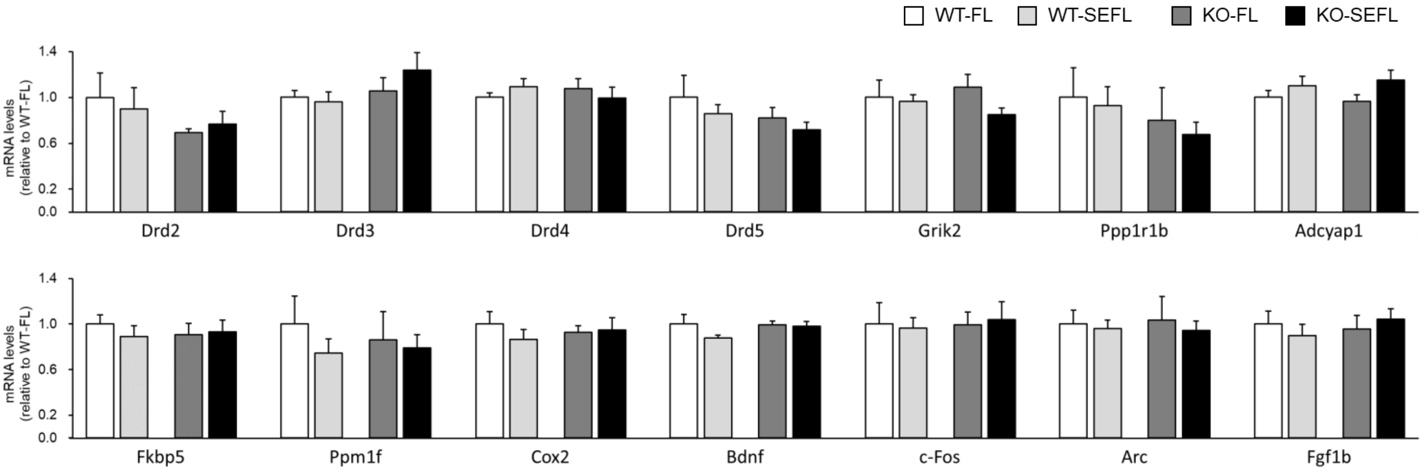

Supplementary Figure 8

A Western blot from ventral HP (naïve mice)

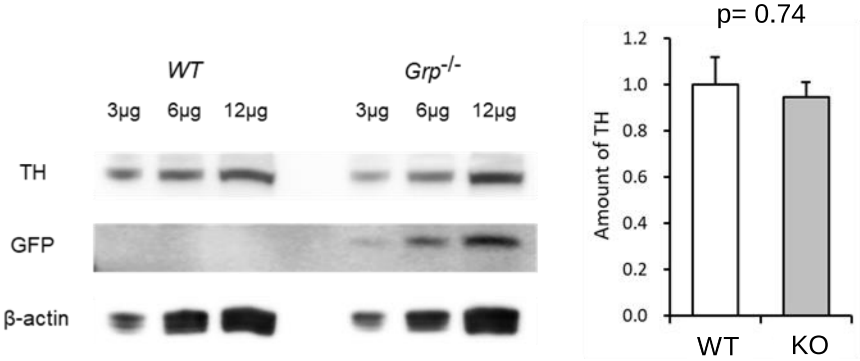

B BLA qPCR (naïve mice)

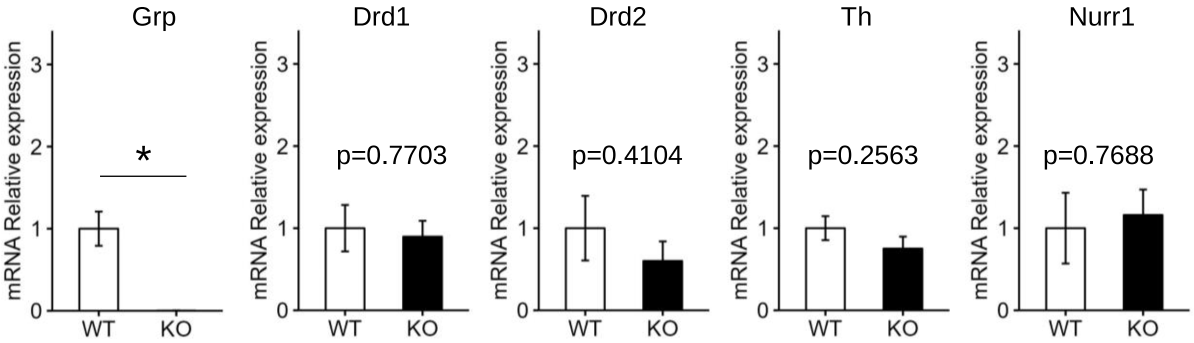

C VTA qPCR (naïve mice)

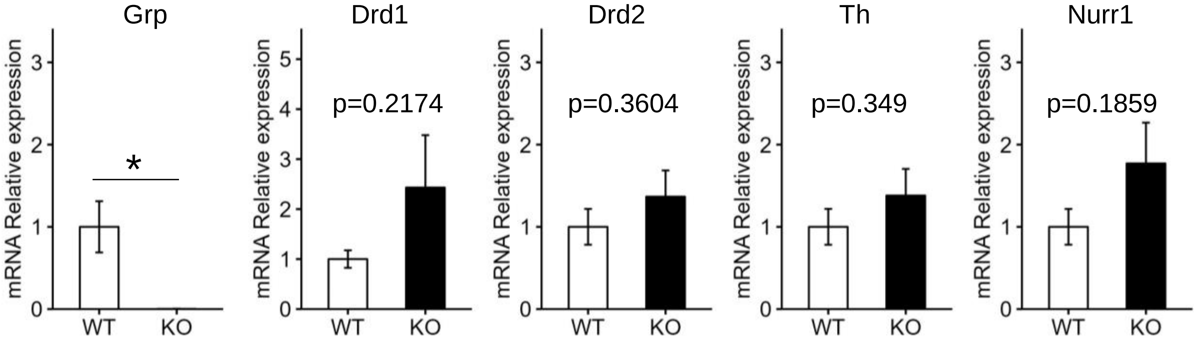

Supplementary Figure 9

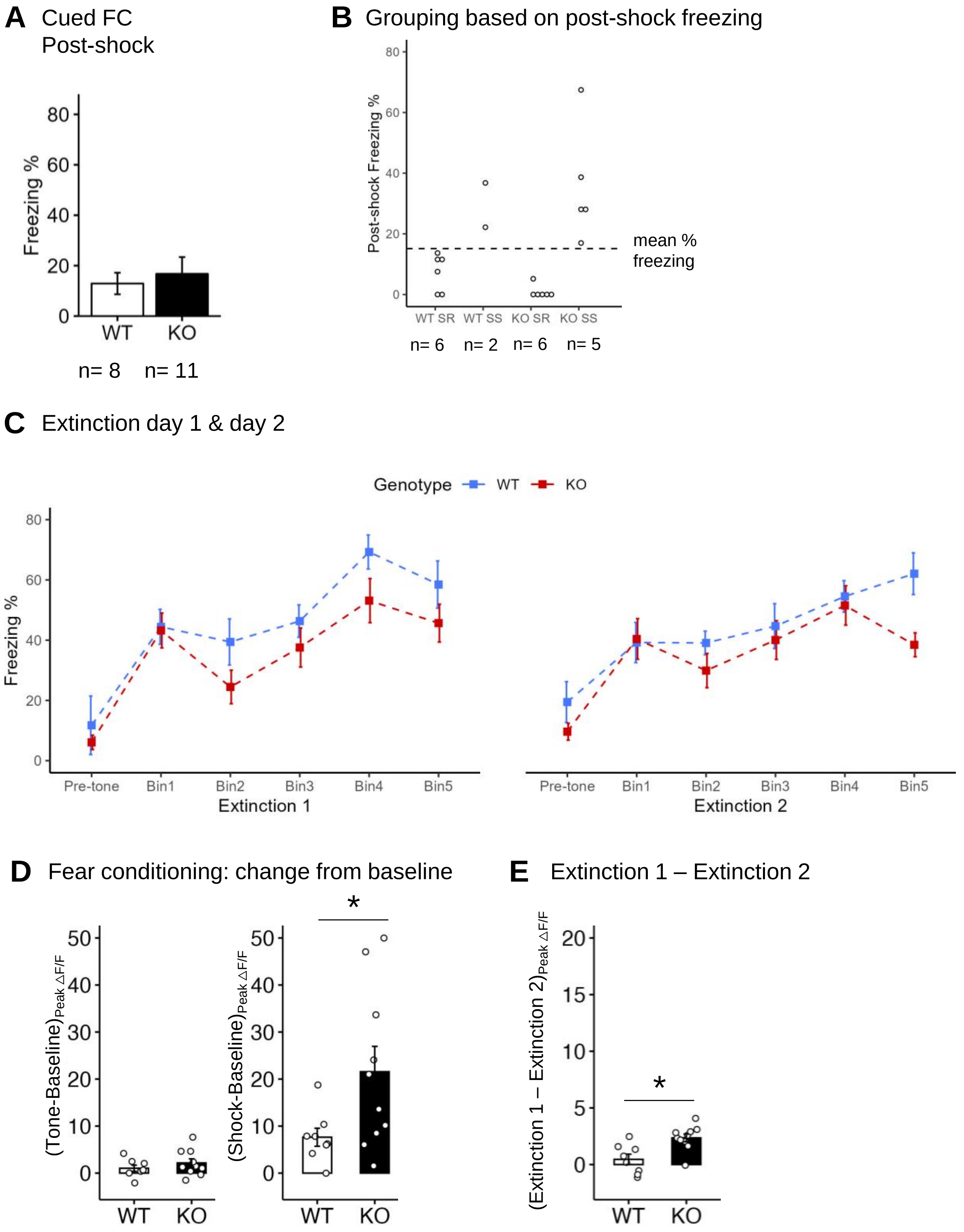

Supplementary Figure 10

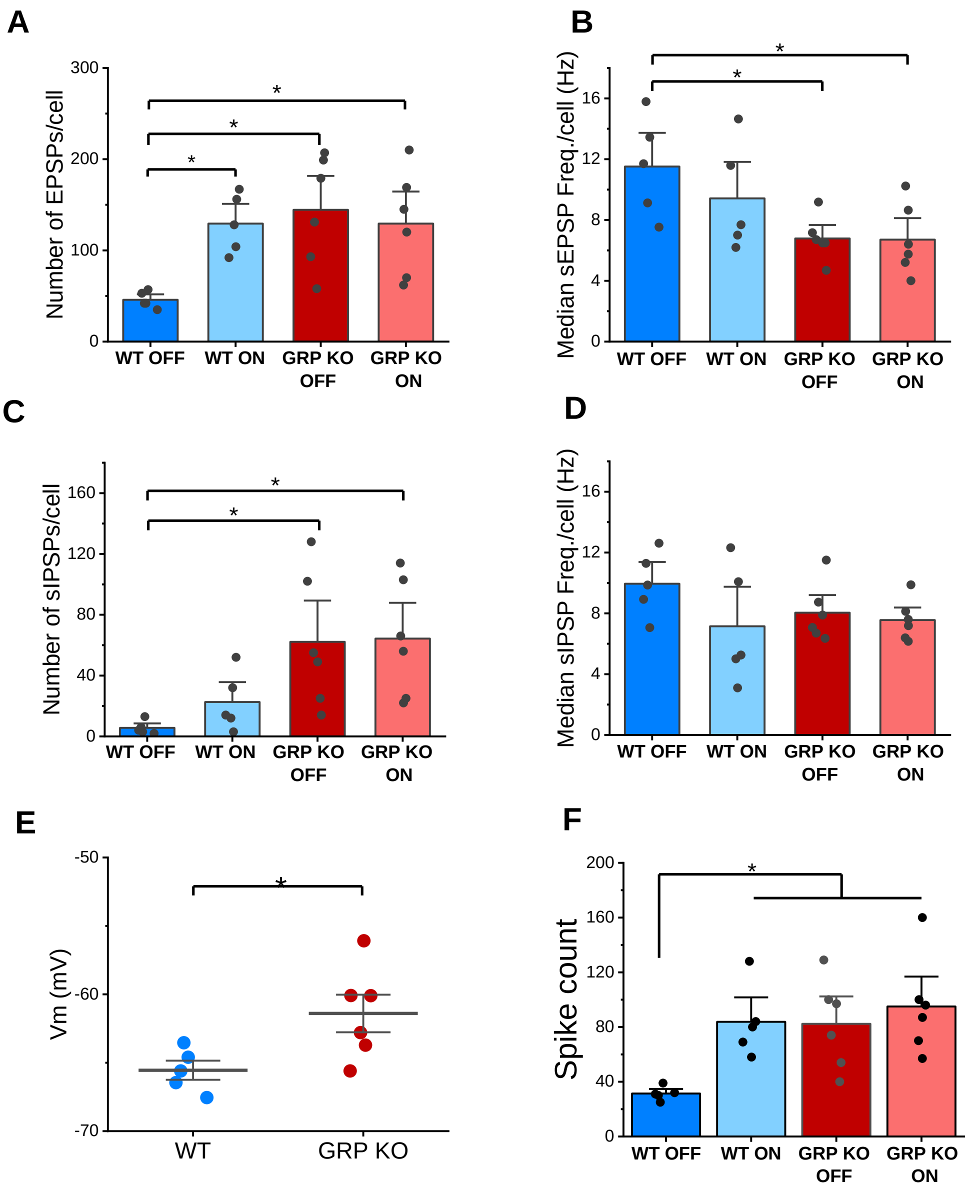

Supplementary Figure 11

A Amygdala RNAseq after the 30-day recall test (genes also tested with qPCR)

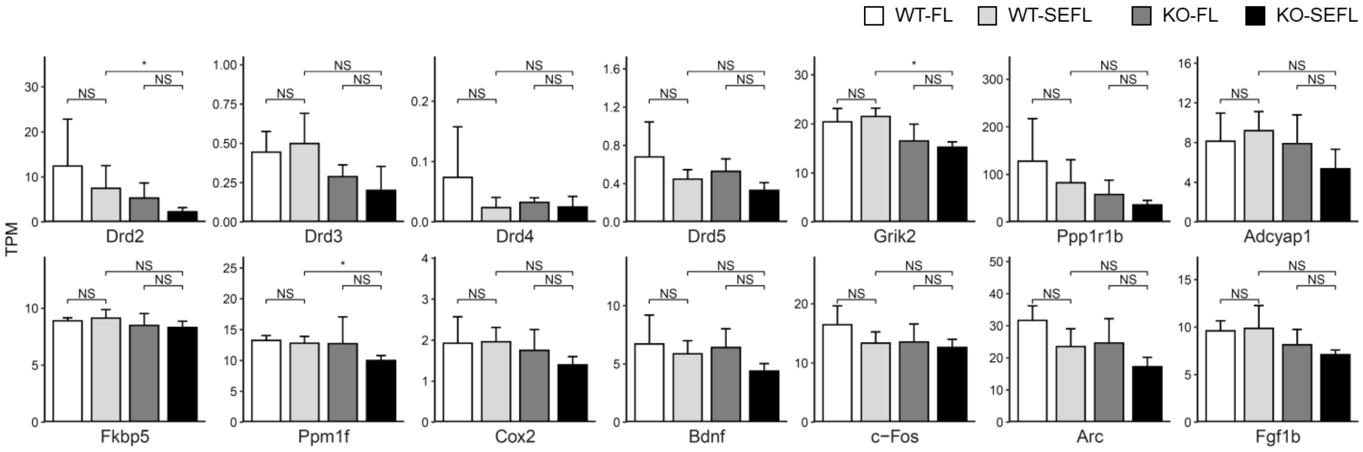

B Volcano plots for Amygdala RNAseq (dopamine-related genes)

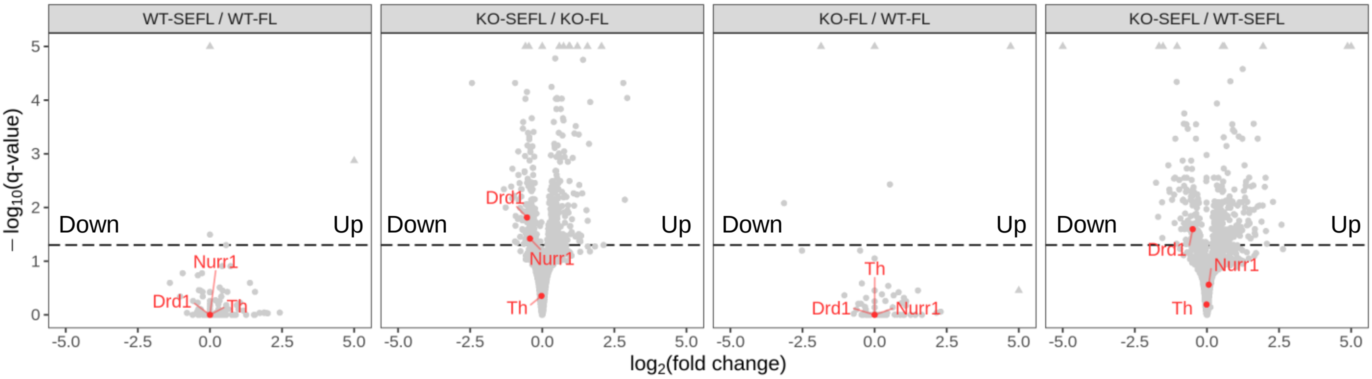

Supplementary Figure 12

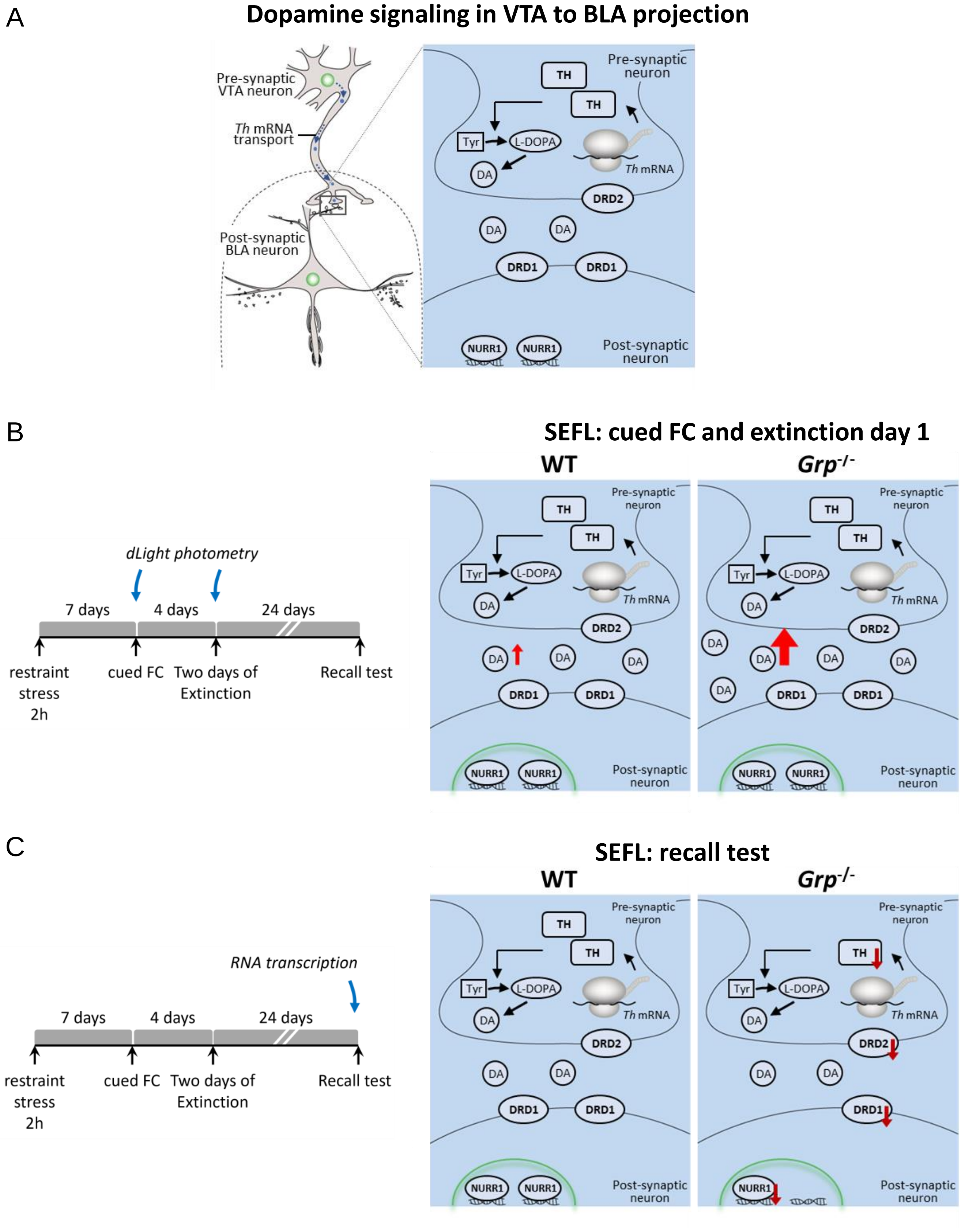

Supplementary Figure 13

Th

Two-way factorial ANOVA	F value	Pr(>F)
Genotype	17.539	<0.001 *
Stress	16.928	<0.001 *
Batch	0.954	0.333
Genotype:Stress	8.420	0.0053 *
Genotype:Batch	0.032	0.860
Stress:Batch	2.914	0.094

Nurr1

Two-way factorial ANOVA	F value	Pr(>F)
Genotype	0.457	0.502
Stress	5.437	0.0235 *
Batch	1.716	0.196
Genotype:Stress	0.515	0.476
Genotype:Batch	3.810	0.056
Stress:Batch	0.163	0.688

Bonferroni's correction	P value
WT-FL vs WT-SEFL	0.220
KO-FL vs KO-SEFL	0.041*

Drd1

Two-way factorial ANOVA	F value	Pr(>F)
Genotype	19.112	<0.001 *
Stress	0.010	0.921
Batch	1.600	0.211
Genotype:Stress	0.376	0.542
Genotype:Batch	0.005	0.944
Stress:Batch	0.734	0.396

Bonferroni's correction	P value
WT-FL vs KO-FL	0.0051 *
WT-SEFL vs KO-SEFL	<0.001 *

Supplementary Table S1

SUPPLEMENTARY INFORMATION

Dopaminergic signaling is modulated in gastrin-releasing peptide neurons in opposite directions during stress-enhanced fear learning and extinction

Yoshikazu Morishita, Ileana Fuentes, Sofia Gonzalez-Salinas, John Favate, Jennifer Mejaes, Ko Zushida, Akinori Nishi, Charles Hevi, Noriko Goldsmith, Steve Buyske, Stephanie E. Sillivan, Courtney A. Miller, Eric R. Kandel, Shusaku Uchida, Premal Shah, Juan Marcos Alarcon, David Barker, Gleb P. Shumyatsky

Materials / Subjects and Methods

Mice. Generation of the *Grp*$^{-/-}$ mice (***Supplementary Figure 1***): The portion of the mouse *Grp* gene 4.5 kb long that includes exon 1 was isolated, most of exon 1 was deleted and replaced with the *EGFP* cDNA (Clonetech; Mountain View, CA), inserted into the plasmid pnCreFNF14.19 (after removing nCre cDNA; gift from Xiaoxi Zhuang and Rene Hen). The ES work and chimera generation were performed according to standard methods using the 129 strain. The *Grp*$^{-/-}$ mice were backcrossed and maintained on C57BL/6J background (N>10). The homozygous *Grp*$^{-/-}$ mice and their wildtype littermates were generated by breeding heterozygous *Grp*$^{+/-}$ mice, which in turn resulted from breeding heterozygous mice to C57BL/6J mice (Jackson Laboratory; Bar Harbor, ME). All mice were maintained on a 12-h light/dark cycle. Mice were housed, 4–5 per cage, in a 12-hour (6 AM to 6 PM) light/dark colony room at 22C. Male mice were used in all experiments. Food and water were provided ad libitum. Behavioral experiments were conducted during the light phase of the cycle, and mice were at least 12 weeks old at the time of training. Sample size was chosen based on previous publications of our group using similar procedures. WT and KO mice were always included in each session. The order of genotypes was intermixed, but no randomization method was used.

Immunohistochemistry. Immunohistochemistry was performed as previously described (1-3). Mice were deeply anesthetized with avertin (250 mg/kg i.p.) and transcardially perfused with 4% PFA. Their brains were postfixed overnight in 4% PFA and cryoprotected in 30% sucrose. The brains were sectioned (40 µm) using a cryostat, and single or double immunofluorescence was performed on free-floating sections. Primary antibodies were rabbit polyclonal antibody against green fluorescent protein (GFP, 1:500; Cat. A11122 Invitrogen; Waltham, MA); chicken antibody against GFP (Cat. A10262, 1:1000, Invitrogen), or rabbit anti-Tyrosine hydroxylase (Cat. Ab152, 1:500, MilliporeSigma; Rockville, MD). Secondary antibody was conjugated with AlexaFluor-488 (anti rabbit 1:500, Cat. A11008; anti chicken 1:500, Cat. A11039; Invitrogen) or AlexaFluor-594 (anti rabbit 1:500, Cat. A11012, Invitrogen). Images were acquired using an LSM 510 META laser confocal microscope or Observer Z1 (Zeiss; White Plains, NY) with multichannel excitation and detection options, including optimal factory-recommended filter configurations to minimize spectral bleed-through.

rAAV2-based retrograde tracing. For virus injections, mice were anaesthetized intraperitoneally with avertin (250 mg/kg) and placed in a stereotaxic frame. The skull was exposed and a small portion of the skull over dorsal hippocampus was removed bilaterally with a drill. Subsequently, AAV vectors (rAAV2-CaMKII-tdTomato (4); 1.0×10^{13} viral genomes per ml) dissolved in physiological saline were injected bilaterally into the LA (AP: -2.0 mm, ML: ±3.25 mm, DV: -4.25 mm) or BA (AP: -1.5 mm, ML: ±2.8 mm, DV: -4.75 mm) in 0.25 µl volume. The needle was slowly withdrawn 5 min after the injection. Mice were perfused 3 weeks after surgery, and the brains were sectioned at 40 µm. Anti-GFP immunofluorescence was performed on free-floating sections. The percentage of the GRP-positive cells expressing the retrograde tracer (*rAAV2-retro-CaMKII-tdTomato*) versus the total number of the GRP-positive cells in each brain region was quantified using the ImageJ Cell Counter plugin.

Fear conditioning. Contextual and cued fear conditioning was performed as described previously (5). Mice were individually housed for 7 days prior to the behavioral test. During training, a mouse was placed in the conditioning chamber (Med Associates; Fairfax, VT) for 120 s before the onset of the neutral tone that lasted for 30 s at 2800 Hz, 85 dB. The last 2 s of the tone were paired with the unconditioned stimulus (US), 0.7 mA of continuous foot shock. Thus, the previously neutral tone became conditioned stimulus (CS). After an additional 30 s in the chamber, the mouse was returned to its home cage. To test contextual fear conditioning, 1 hour (STM) or 24 hours (LTM) after training, the mouse was placed back in the training chamber. To test cued fear conditioning, 3 hours following the contextual fear test, the mouse was placed in a novel environment in which the tone (120 s) that had been presented during training was given after a 1-min habituation period (pre-CS). The time spent freezing was assessed with FreezeFrame software (Actimetrics; Lafayette, IN).

Pain sensitivity test. Response to the electric shock was assessed with naïve mice as described previously (6, 7). The intensity of the shock required for running, vocalization, and jump was determined for each mouse by delivering a 1-s-long shock every 30 s starting at 0.08 mA and increasing the shock 0.02 mA each time. Testing was stopped after all behaviors were noted.

Open field test. This test was performed as previously reported (8). The open field consisted of a white arena (43.2 cm × 43.2 cm × 40 cm) coupled to an automated video tracking system (Open Field Activity Software, Med Associates). Mice were placed in the corner of the arena, and the time spent in the center area and the total distance traveled (locomotion) were measured.

Elevated plus maze. This test was performed as previously reported (8). The elevated plus maze (1 m above the floor) consisted of a center platform (5 cm × 5 cm), two open arms (40 cm × 5 cm), and two closed arms (40 cm × 5 cm) within walls (height 30 cm). Mice were placed individually in the center of the apparatus, and the time spent in each arm was measured for 10

min using Limelight software (Actimetrics). Results are expressed as the percentage of the time spent in closed arms over the total time spent in the maze.

Light-Dark transition test. A light/dark transition test was conducted as previously described (9). The apparatus used for this test comprised a cage (43.2 cm × 43.2 cm × 40 cm) divided into two sections of equal size by a partition. One chamber was brightly illuminated, whereas the other chamber was dark. Mice were placed into the dark side of the cage at the start of the experiment and allowed to move freely between the two chambers for 10 minutes. The distance travelled in each chamber and time spent in each chamber were recorded using automated video tracking system (Open Field Activity Software, Med Associates).

Bombesin-saporin injections in the BLA. Mice were anaesthetized intraperitoneally with avertin (250 mg/kg) and placed in a stereotaxic frame. The skull was exposed and a small portion of the skull over the BLA was removed bilaterally with a drill. Subsequently, bombesin-saporin or blank-saporin (80 ng/μl) (10) dissolved in saline were injected bilaterally into the BLA (AP: -2.0 mm, ML: ±3.25 mm, DV: -4.3 mm) in 0.3 μl volume. The needle was slowly withdrawn 5 min after the injection. Mice were trained in cued-fear conditioning at 2 weeks after surgery. On the training day, each mouse was placed in the conditioning chamber (Med Associates) for 120 s before the onset of the CS, a tone that lasted for 30 s at 2800 Hz, 85 dB. The last 2 s of the CS was paired with the US, a 0.7 mA of continuous foot shock. After an additional 30 s in the chamber, the mouse was returned to its home cage. 24 hours after training, mice were placed in a novel environment in which the tone (120 s) that had been presented during training was presented after a 1 min habituation period (pre-CS). The time spent freezing was assessed using FreezeFrame software (Actimetrics software). At the end of the experiment animals were perfused transcardially with ice-cold solution of 4% paraformaldehyde and brains were processed for in-situ hybridization against the GRPR as described (6).

SEFL paradigm. SEFL is a behavioral assay that consists of an acute restraint stress followed by fear conditioning and extinction, as we described before (11). Each genotype was divided into two groups: Stress-Enhanced Fear Learning (SEFL) and Fear Learning (FL). As shown in (**Figure 2E**), the SEFL group underwent the restraint stress by placing each mouse into a clear 50 ml conical centrifuge tube (Falcon) with ventilation holes for 2 hours. Tubes were placed flat in an open box in a biosafety cabinet with overhead lights on for the duration of the procedure. The FC control group did not undergo the restraint stress: each mouse was instead placed in a biosafety cabinet in another room and briefly handled in its home cage. 7 days after the restraint stress of the SEFL group, mice in both SEFL and FL groups were habituated to the training context A three times a day for a total of 12 minutes. 24 hours later, mice underwent a mild protocol of fear conditioning: 2 minutes of exploration followed by two 30 second CS-US pairings that co-terminated with a 0.5 mA footshock (US) separated by a 60- or 120-second intertrial interval (ITI; both produce the same results). The CS was an 85 dB, 10 kHz tone. This mild fear conditioning protocol was employed to avoid a ceiling effect and the potential for induction of a depressive-like phenotype in stressed animals. Mice were removed from the training context 1 minute after the second shock and immediately returned to their home cages. Context A consisted of grid floors, a dim corner light in the room, no overhead lights and 70%

ethanol used for cleaning. Extinction training (4 days post shock) and remote memory retrieval tests (Recall; 30 days post shock) were performed in novel context B, consisting of smooth plastic flooring, a plastic insert on the walls of the chamber, bright overhead lights, chamber lights on, orange scent, a 65 dB white noise and isopropanol for cleaning. Following a 2-minute exploration in Context B, animals were given 30 (extinction) or 5 (recall) CS presentations in the absence of the US (tone only), each separated by a 60 second ITI. The brains from animals used to generate the SEFL behavioral data in ***Figure 2F-I*** were used for qPCR (***Figure 3A***) or RNA-seq (***Figure 6***). Also, the SEFL behavioral data corresponding to dLight experiment (***Figure 4***) are now shown in ***Supplementary Figure 10***.

dLight Fiber Photometry. Mice were anesthetized with Avertin (250 mg/kg) and secured to a stereotaxic frame. After exposing the top of the skull, the mouse's head was leveled to ensure that the dorsoventral difference between bregma and lambda was < 100μm. AAV-hSyn-dLight1.2 (12) was then injected into the BA (0.2 μl; AP: -1.8mm, ML: ± 3.2 mm, DV: -4.8 mm) using a 2 μL-syringe (Cat. 7002, Hamilton; Reno, NV) and an infusion pump (Cat.53311, Stoelting; Wood Dale, IL)**.** The human synapsin (hSyn) promoter is widely used because it directs strong and highly neuron-specific long-term transgene expression and is one of the most ubiquitous promoters in current use for experiments in brain tissue (13, 14). Our goal in the dLight photometry experiments was to see overall BLA responses, and thus we decided to use a widely expressing promoter. The injector was left in place for 10 minutes following the end of the injection to minimize diffusion. Subsequently, a 400 μm core optic fiber embedded in a 2.5mm ferrule (Doric Lenses; Quebec, Canada) was implanted 100 μm above the same target site and secured to the skull using dental cement and a set of 2 jewelers screws. Following surgery mice recovered on a heating pad before being transferred to the colony. The mice remained in recovery for 2 weeks to allow for virus expression prior to the start of the SEFL protocol and fiber photometry recordings. For all recordings, dLight was excited at two wavelengths (470 nm, calcium-dependent signal and 405 nm isosbestic control) by amplitude modulated signals from two light-emitting diodes that were coupled into a 400 mm 0.48NA optic fiber. Signals emitted from dLight and its isosbestic control channel then filtered through a 500-550 nm emission filter and recorded using a Doric Fluorescence Detector. Signals were digitized at 1 kHz by a real-time signal processor (RZ5D; Tucker Davis Technologies; Alachua, FL) running the Synapse software suite. Analysis of the resulting signal was then performed using custom-written MATLAB scripts (https://github.com/djamesbarker/FiberPhotometry). Changes in fluorescence across the experimental session (DF/F) were calculated by smoothing signals from the isosbestic control channel (15), scaling the isosbestic control signal by regressing it on the smoothed dLight signal, and then generating a predicted 405 nm signal using the linear model generated during the regression. dLight independent signals on the predicted 405 nm channel were then subtracted from the raw dLight signal to remove movement, photo-bleaching, and/or fiber bending artifacts. Signals from the dLight channel were then divided by the control signal to generate the DF/F. Peri-event histograms were then created by averaging changes in fluorescence (DF/F) across repeated trials during windows encompassing behavioral events of interest. Signals for each mouse were then normalized using a robust z-score by subtracting the median of a 5 s window just before the peri-event histogram from each sample and then dividing the entire sample by the median absolute deviation. The Peak and Area Under the Curve (AUC) are then calculated by finding the local maxima (Peak) or by taking the integral over the time periods defined for the cue and shock

(AUC), respectively. Freezing behavior was analyzed using the Freeze Analysis Module of EzTrack (16). At the end of the experiments, viral expression was confirmed by immunohistochemistry anti-GFP antibodies, and the locations of the fibers were verified.

***ex vivo* Electrophysiology of VTA-> BLA circuit.** Mice were anaesthetized with isoflurane gas anesthesia (1-3.5%) and placed in a stereotaxic frame. The skull was exposed, and two incisions were made over the VTA (AP: -3.2 mm, ML: ±0.3 mm, DV: -4.5 mm). Subsequently, AAV8-hSyn-ChR2-GFP (1.0×10^{13} viral genomes per ml) dissolved in saline (0.3 µl/side) was injected into the VTA at a 0.1 µl/min. The needle was slowly withdrawn 5 min after the injection. For electrophysiology, mice were deeply anesthetized with vaporized isoflurane (5% in 100% oxygen for 3 min) and immediately euthanized by rapid decapitation. Brains were dissected quickly and chilled in ice-cold dissection artificial cerebrospinal fluid (dACSF containing: (mM) 110 Choline-Cl, 2.5 KCl, 7 MgSO4, 0.5 CaCl2, 25 NaHCO3, 1.25 NaH2PO4, 3.1 Na-pyruvate, 5.8 Na-ascorbate and 25 Glucose saturated with 95% O2, 5% CO2). Transverse slices (300 µm) containing the amygdala were prepared with a vibratome 3000 in dACSF and collected into recording ACSF (rACSF containing (mM) 125 NaCl, 2.5 KCl, 1 MgSO4, 2 CaCl2, 25 NaHCO3, 1.25 NaH2PO4, 2 Na-pyruvate and 25 Glucose saturated with 95% O2, 5% CO2) and then warmed in oxygenated rACSF to 35°C for 45 min. Slices were thereafter allowed to equilibrate for at least 45 min in oxygenated rACSF at room temperature. For experiments, slices were placed in a submerged recording chamber continuously perfused with oxygenated rACSF at 35-36°C at the rate of 1 ml/min. For whole-cell recordings, the patch electrodes (5–10 MΩ; Sutter Inst. Co., Novato, CA. USA) were filled with pipette solution containing 120 mM K-gluconate, 10 mM NaCl, 5 mM MgSO4, 5 mM HEPES, 0.6 EGTA, 2 mM NaATP, 0.3 mM NaGTP, 10 mM phosphocreatine (290 mOsm, adjusted to pH 7.3 with KOH). Cells were visually identified with an inverted fluorescence microscope (Olympus BX51WIF). Whole-cell patch clamp recordings were obtained using a Multiclamp 800B amplifier and acquired using the pClamp 10 software (Molecular Devices; San Jose, CA). After formation of a gigaohm seal, whole-cell configuration was attained. For measurements of resting membrane potential and synaptic potentials, the recording was switched to bridge and current-clamp mode, respectively. All data were sampled at 10 kHz, filtered at 5 kHz, and recorded onto computer. Offline data analysis was performed using Clampfit 10 program (Molecular Devices). Photostimulation: Blue collimated light-emitting diode (LED) with 455 nm peak wavelength (FC2-LED-455Z-540HR, Prizmatix; Southfield, MI) was used for photostimulation of ChR2-expressing VTA fibers. The LED was connected to a Master-8 Pulse Stimulator through the LED driver. Brain slices in recording chamber were illuminated through 40x water-immersion objective lens (IR-DIC, Olympus). VTA fibers were identified via fluorescent stimulation with excitation (494 nm) and emission (518 nm) filters (FITC filter set, Olympus BX51WIF) and detected with a ThorsLab fluo camera (1500M-GE; Newton, NJ). Illumination area covered approximately 0.25 mm^2 with the soma of the neuron patched for recording at the center. To evoke synaptic responses in the amygdala by photostimulation of VTA fibers, we illuminated slices with blue light pulses of 10 ms duration at 50 Hz for 60 s. Data are presented as mean ± SEM, and statistical comparisons between groups were performed with one-way ANOVA tests using the Origin 8.0 software (Origin Labs; Northampton, MA).

RNA isolation and cDNA synthesis. Separate groups of animals were used to isolate RNA for qPCR and RNA-seq analyses. These two experiments were separated in time by several months. The amygdala was dissected as previously reported. In brief, mouse brains were immediately extracted and put on ice. Bilateral punches of the amygdala (preferentially including BLA) were obtained. Collected tissue was immediately put into RNA later (QIAGEN; Germantown, MD) until processing. Total RNA from dissected tissues was extracted by using the RNeasy Mini Kit (QIAGEN). For RNA-seq, total RNA was sent to the company, which made the libraries, and the RNA-seq were performed on Illumina Novaseq6000 (Novogene; Sacramento, CA). For qPCR, one microgram of total RNA was used for cDNA synthesis by SuperScript IV Reverse Transcriptase (Invitrogen). The cDNA was stored at -80°C until use.

Quantitative real-time PCR. Real-time PCR was performed using the Applied Biosystems 7900HT Fast Real-Time PCR System with SYBR green PCR Master Mix (Applied Biosystems) according to the manufacturer's protocol. PCR conditions were 10 min at 95°C, followed by 40 cycles at 95°C for 15 sec and 60°C for 30 sec. Amplification curves were visually inspected to set a suitable baseline range and threshold level. The relative quantification method was employed according to the manufacturer's protocol in which all target mRNA expression levels were normalized to *Gapdh* expression.

RNA-seq and data analysis. We used Novogene to perform Ribo-Zero™ & NEBNext Ultra™ II Directional RNA Library Prep Kit. Ribosomal RNA was removed by rRNA removal kit, and rRNA free residue was cleaned up by ethanol precipitation. The length of sequencing reads was 150 bp paired end reads and it was done on Illumina NovaSeq. The number of replicates used for sequencing was 4.

Data analysis: Adapter removal and quality trimming of raw data was performed with fastp (18). Processed reads were then aligned with kallisto (19) to the gencode vM25 mouse transcript sequences (20). TPMs were reevaluated for each sample by first rounding the number of reads mapping to each transcript, then recalculating TPM. When gene level TPMs are presented, they are the sum of TPMs from each isoform of a gene. All analysis and graphs were produced with the R programming language (21) and the tidyverse set of packages (22). tximport (23) was used to summarize isoform level counts to the gene level and the results were passed to DESeq2 (24) for differential expression. We used for the DEG analysis q-values, i.e. p-values that were corrected for multiple hypothesis testing; the "apeglm" method (25) was used for shrinkage of fold-changes. GO analyses were performed by submitting genes ENSEMBL IDs to the online PANTHER webform (http://pantherdb.org/) using the statistical overrepresentation test and FDR p-value adjustment (26, 27). A single submission was made for each of the 4 sets of differentially expressed genes, namely up or downregulated KO SEFL / KO FL and up or downregulated KO SEFL / WT SEFL. No significant categories were found using downregulated genes from KO SEFL / WT SEFL.

Western blotting. Western blotting was performed (3) using equal amounts of protein separated on 12% Bis-Tris gels (Life Technologies) and transblotted onto polyvinylidene difluoride membranes (GE Healthcare Bio-Sciences). After blocking with 5% skim milk, the

membranes were incubated with anti-Tyrosine Hydroxylase antibody (Cat. Ab152, MilliporeSigma) or anti-GFP Polyclonal antibody (Thermofisher; Waltham, MA). After incubation with HRP-linked anti-rabbit IgG, the blots were developed using the ECL-Plus Detection Kit (GE Healthcare Bio-Sciences; Chicago, IL). Densitometric analysis was performed using ImageQuant software (GE Healthcare) after scanning (Kwikquant imager, Kindle Biosciences; Greenwich, CT).

Data analyses and statistics. Analyses of the data were performed using an appropriate ANOVA. Significant effects were determined using Fisher's post hoc test or Bonferroni's correction. Unpaired Student's t tests were used for two-group comparisons. In all cases, p values were two-tailed, and the comparisons were considered to be statistically significant when $p < 0.05$. All data are presented as the mean ± SEM. MATLAB and R were used for the analyses. The investigators were blind to the genotype or treatment of the mice during experimentation.

Data availability. All the data used in this study are included within the manuscript's figures or provided in the supplementary information section and Source Data files. The raw sequencing data are deposited under the GEO accession GSE164226. Any additional data and information are available upon request to the corresponding authors, Drs. David Barker, Premal Shah, Juan Marcos Alarcon and Gleb P. Shumyatsky. Source data are provided with this paper.

Code availability. Scripts created to process and analyze RNAseq data are available upon request.

FIGURE LEGENDS

Supplementary Fig. 1. Generation of the *Grp*$^{-/-}$ mouse. (A) Diagram illustrating the *Grp* gene targeting design. PGK-neomycin was removed by crossing with CAG-FLPe mouse. The arrows show primers for PCR genotyping. (B) The underlined part shows the DNA sequence deleted by targeting. (C) The following primers were used for genotyping PCR (WT F: GG ACAACGCACTCTCAGCCTAGT, WT R: AGACGGGGCTCCCTCTAGCTAG, KO R: ACTGGGTGCTCAGGTAGTGGTTGT). (D) The *Grp*$^{-/-}$ mouse shows no obvious anatomical abnormalities. Histology of the basolateral amygdala and ventral hippocampus in adult (3 months old) wildtype and *Grp*$^{-/-}$ mice. Consecutive 40-µm coronal sections were collected and stained for NeuroTraceTM 530/650 (1:100).

Supplementary Fig. 2. Schematic of retrograde tracing by AAV injection. Retrograde rAAV2-retro-CaMKII-tdTomato was injected into the BLA or mPFC of *Grp*$^{-/-}$ mouse brain. Three weeks following injections, the mice were perfused, and the brains were coronally sectioned at a thickness of 40 µm. (A) Schematic diagram of the virus injection. (B) rAAV2-retro-CaMKII-tdTomato was injected into LA. There were tdTomato-positive cells in the MGm/PIN, but there were no cells colocalized with GFP.

Supplementary Fig. 3. The *Grp*$^{-/-}$ mice and GRP/GRPR double knockout mice are normal in post-shock freezing. (A) *Grp*$^{-/-}$ mice post-shock freezing (p=0.953; WT n=21, *Grp*$^{-/-}$ n=22) for long-term memory experiment. (B) *Grp*-/- mice post-shock freezing (p=0.902; WT n=10, *Grp*$^{-/-}$ n=9) for short-term contextual memory experiment. (C) Grp -/- mice post-shock freezing (p=0.632; WT n=12, *Grp*$^{-/-}$ n=13) for short-term cued memory experiment. (D) GRP/GRPR double knockout mice: WT n=10, DKO n=10; post-shock freezing: p=0.963. Data presented as mean ±SEM.

Supplementary Fig. 4. Transcription of immediate-early genes *c-Fos* and *Arc* in the amygdala following fear conditioning was enhanced in *Grp*$^{-/-}$ mice. (A) Expression levels of c-Fos mRNA in the amygdala of *Grp*$^{-/-}$ mice. (B) Expression levels of Arc mRNA in the amygdala of *Grp*$^{-/-}$ mice. The amygdala tissue was dissected 30 min after fear conditioning. c-Fos and Arc mRNA expression levels were normalized to Gapdh expression and verified by normalization to β-actin. Results are expressed as x-fold change normalized to wildtype naïve controls. Tukey post-hoc test, *c-Fos* p=0.002, *Arc* p=0.011. *p<0.05 vs. respective naive. #p<0.05 vs. compared to wild type. Data presented as mean ±SEM.

Supplementary Fig. 5. *Grp*$^{-/-}$ mice showed normal anxiety and pain sensitivity. (A) Open field test, (B) Elevated plus maze, (C) Light-dark transition test. No difference was found between groups (wildtype mice, n=15; knockout, n=15; OF p=0.205, total distance p=0.785; EPM p=0.740; LD transition p=0.820). (D) Pain sensitivity thresholds. The intensity of shock required to elicit three reactions, movement (movt), vocalization (vocal), and jump, were assessed and data are presented as the mean ±SEM. No difference was found between groups (wildtype, n=6; knockout, n=6; movement p=0.329, vocalization p=0.511, jump p=0.705).

Supplementary Fig. 6. Decrease in freezing during extinction and increase in freezing during Recall. (A-B) To compare the extinction rate among groups, we calculated the percent decrease in freezing by subtracting freezing at bin 5 from freezing at bin 1 for each extinction session. Data were analyzed with a two-way ANOVA. Freezing decrease did not differ between genotypes or stress in either Extinction 1 (Genotype F(1,64)=0.133, p=0.716; Stress F(1,64)=0.049, p=0.826; Genotype X Stress F(1,64)=0.147, p=0.702) or Extinction 2 (Genotype F(1,64)=2.593, p=0.112; Stress F(1,64)=2.085, p=0.154; Genotype X Stress F(1,64)=0.466, p=0.497). (C) Percent of increase in freezing during long-term recall memory. Freezing during the last bin of Extinction 2 was subtracted from freezing during Recall. Data were analyzed with a two-way ANOVA. There were no significant differences for Genotype, Stress or the Interaction (Genotype F(1,64)=0.024, p=0.877; Stress F(1,64)=0.711, p=0.402; Genotype X Stress F(1,64)=0.576, p=0.451). Data are presented as mean ± standard error. (D) dLight basal signal was subtracted from tone or from shock epochs using the Peak △F/F data shown in Figure 4E. t-test, *p<0.05 (E) dLight signal from Extinction 2 was subtracted from Extinction 1 using the Peak △F/F data shown in Figure 4F. t-test, *p<0.05.

Supplementary Fig. 7. Susceptibility comparison between WT and KO mice. Freezing during Extinction 1, Extinction 2, and Recall were compared between Genotypes and Susceptibility groups (SR= Stress Resilient, SS= Stress Susceptible). Extinction data were averaged across the 5 bins for each of the sessions. Data were analyzed with a two-way ANOVA. (A) Freezing during Extinction 1 was significantly affected by Genotype and Susceptibility but not by their Interaction (Genotype $F(1,36)=31.578$, $p<0.01$; Susceptibility $F(1,36)=8.212$, $p=0.00691$; Genotype X Susceptibility $F(1,36)=2.62$, $p=0.114$). (B) Freezing during Extinction 2 was significantly affected only by Genotype (Genotype $F(1,36)= 9.615$, $p=0.00374$; Susceptibility $F(1,36)=3.475$, $p=0.0705$; Genotype X Susceptibility $F(1,36)=1.148$, $p=0.29104$). (C) Freezing during Recall was significantly affected by Genotype only (Genotype $F(1,36)= 13.249$, $p<0.01$; Susceptibility $F(1,36)=0.100$, $p=0.753$; Genotype X Susceptibility $F(1,36)=2.984$, $p=0.093$). These results show that Genotype is consistently contributing to higher levels of freezing during Extinction 1, Extinction 2 and Recall even when mice are sorted out according to their stress susceptibility. Data are presented as mean ± standard error.

Supplementary Fig. 8. Dopamine signaling-related genes, examined by qPCR, are downregulated in *Grp*$^{-/-}$ mice following recall in SEFL. Analysis of dopamine signaling-related genes and stress susceptibility-related genes in the BLA. After the recall test, the mice were returned to their home cage and the amygdala tissue was dissected 30 min later. Quantitative real-time PCR analysis: all target mRNA expression levels were normalized to *Gapdh* expression and verified by normalization to *β-actin*. Results are expressed as x-fold change normalized to wildtype controls. All measurements were performed in triplicate (WT-FL n=5, WT-SEFL n=11, KO-FL n=7, KO-SEFL n=10). For statistics, see Table S1.

Supplementary Fig. 9. Expression of Tyrosine hydroxylase (TH) expression and dopamine-signaling genes is normal in naïve *Grp*$^{-/-}$ mice. (A) Western blot on wildtype and *Grp*$^{-/-}$ mice, using antibodies against TH or GFP (the GFP cDNA is knocked-in into the *Grp* gene in the *Grp*$^{-/-}$ mice). 3, 6 and 12 µg of whole-cell extracts from the ventral hippocampus were separated on 12% Bis-Tris gels, and transblotted onto PVDF membranes. The relative expression level of the TH was normalized to β-actin levels. (B) Quantitative real-time PCR (qPCR) of dopamine signaling-related genes in the BLA (WT n=6, KO n=6). (B) Quantitative real-time PCR (qPCR) of dopamine signaling-related genes in the VTA (WT n=7, KO n=8). qPCR data were normalized to gapdh expression and are plotted as fold change from WT. Data were analyzed with a Student's t-test. *$p<0.05$.

Supplementary Fig. 10. Behavioral data during dLight recordings. (A) Post-shock freezing was not significantly different between genotypes ($p=0.632$, t-test). (B) Mice were separated into Stress Resilient (SR) and Stress Susceptible (SS) according to the mean freezing during post-shock freezing. The SS/SR ratio was higher in *Grp*$^{-/-}$ as compared to WT mice. (C) Freezing during extinction did not differ between genotypes (Time $F(1, 179)= 25.66$, $p<2X10^{-16}$; Genotype X Time $F(11,179)=1.101$, $p=0.363$; Genotype $F(1,179)=0.015$, $p=0.902$). (D) Baseline dLight signal was subtracted from signal during Tone presentation ($p=0.32$, unpaired t-test) or from signal during Shock presentation ($p=0.033$, unpaired t-test). Data taken from Fig. 4E (E) dLight

signal during Extinction 2 was subtracted from signal during Extinction 1 (p=0.005, unpaired t-test). Data taken from Fig. 4F.

Supplementary Fig. 11. Optogenetic stimulation of VTA-BLA terminals mimics GRP KO's exacerbated expression of sEPSPs but not of sIPSPs. (A) Average number of sEPSP events in recorded BLA neurons. Two-way ANOVA, Genotype F(1,21)=6.07, p=0.024; Optogenetic F(1, 21)=2.92, p=0.104; Genotype X Optogenetic F(1, 21)=6.09, p=0.024. (B) Median sEPSP frequency in recorded BLA neurons. Two-way ANOVA, Genotype F(1,21)=10.31, p=0.0048; Optogenetic F(1, 21)=0.89, p=0.36; Genotype X Optogenetic F(1, 21)=3.93, p=0.025. *Tukey: WT OFF x GRP KO OFF, p= 0.045; WT OFF x GRP KO ON, p= 0.040. All other comparisons p>0.05. (C) Average number of sIPSP events in recorded BLA neurons. Two-way ANOVA, Genotype F(1,21)=12.60, p=0.002; Optogenetic F(1, 21)=0.47, p=0.49; Genotype X Optogenetic F(1, 21)=0.29, p=0.59. *Tukey: WT OFF x GRP KO OFF, p= 0.044; WT OFF x GRP KO ON, p= 0.035. All other comparisons p>0.05. (D) Median sIPSP frequency in recorded BLA neurons. Two-way ANOVA, Genotype F(1,21)=0.52, p=0.47; Optogenetic F(1, 21)=2.50, p=0.13; Genotype X Optogenetic F(1, 21)=1.24, p=0.27. All Tukey comparisons p>0.05. (E) Resting membrane potential (Vm) from recorded WT (blue) and GRP KO neurons (red). Two Sample t-test, t(9)=-2.52, p=0.03. (F) Spike count in recorded BLA neurons. Two-way ANOVA, Genotype F(1,21)=6.44, p=0.021; Optogenetic F(1, 21)=7.06, p=0.016; Genotype X Optogenetic F(1, 21)=2.63, p=0.122. *Tukey: WT OFF x WT ON, p= 0.043; WT OFF x GRP KO OFF, p= 0.039; WT OFF x GRP KO ON, p= 0.0086. All other comparisons p>0.05.

Supplementary Fig. 12. (A) Expression differences in RNA-seq data recapitulate patterns observed in qPCR datasets, but some genes (*Drd2*, *Grik2* and *Ppm1f*) show significant differences in RNA-seq but not in qPCR. Comparisons of TPM (transcripts per million) based on 4 replicates and (*) indicates significant differences based on p-values <0.05 based on a t-test. (B) Volcano plot of differentially expressed genes based on RNA-seq datasets. 3 dopamine signaling-related genes are highlighted in red. The triangles are points where the fold-change or q-value (Benjamini-Hochberg corrected p-values of t-tests), or both, are larger than indicated and have been reduced to aid in visualization. Namely, if the fold change was >5 or <-5, it has been changed to those values, and if -log10(q)>5, it was reduced to 5.

Supplementary Fig. 13. A possible relationship between the GRPergic circuit and dopaminergic circuit in stress-enhanced fear learning (SEFL). (A) A proposed dopamine signaling between the VTA presynaptic and BLA postsynaptic neurons in naïve WT mouse that can be utilized during SEFL. (B) The *Grp* gene knockout may affect the VTA dopaminergic projections to the BLA leading to an initial increase in dopamine release in the amygdala during shock presentation during the fear-conditioning phase and on day 1 of the extinction phase in SEFL. (C) Later the lack of the GRP leads to a compensatory decrease in dopamine-related gene expression in the BLA during the recall phase in the $Grp^{-/-}$ mice, inducing their susceptibility to SEFL (increased fear responses).

Supplementary Table 1. mRNA expression of several genes involved in the dopamine signaling was differentially expressed in KO-SEFL as shown by two-way ANOVA followed by post-hoc test. Total number of each group (WT-FL n=12, WT-SEFL n=18, KO-FL n=13, KO-SEFL n=18) consists of two batches (1st batch: WT-FL n=5, WT-SEFL n=11, KO-FL n=7, KO-SEFL n=10, 2nd batch: WT-FL n=7, WT-SEFL n=7, KO-FL n=6, KO-SEFL n=8).

Supplementary Table 2. This table contains the transcript abundances in transcripts per million (TPM) for each of the samples. It is located in the “data frames” folder inside of the zip file included as supplemental material with this manuscript. The TPMs in this file were recalculated after rounding the read counts.

Supplementary Table 3. This table contains the read counts for each of the samples. It is located in the “data frames” folder inside of the zip file included as supplemental material with this manuscript. Read counts in this file are rounded to the nearest integer.

Supplementary Table 4. This table contains the results of differential expression using DESeq2. It is located in the “data frames” folder inside of the zip file included as supplemental material with this manuscript.

1. Uchida S, Martel G, Pavlowsky A, Takizawa S, Hevi C, Watanabe Y, et al. Learning-induced and stathmin-dependent changes in microtubule stability are critical for memory and disrupted in ageing. Nat Commun. 2014;5:4389.
2. Martel G, Uchida S, Hevi C, Chevere-Torres I, Fuentes I, Park YJ, et al. Genetic Demonstration of a Role for Stathmin in Adult Hippocampal Neurogenesis, Spinogenesis, and NMDA Receptor-Dependent Memory. J Neurosci. 2016;36(4):1185-202.
3. Uchida S, Teubner BJW, Hevi C, Hara K, Kobayashi A, Dave RM, et al. CRTC1 Nuclear Translocation Following Learning Modulates Memory Strength via Exchange of Chromatin Remodeling Complexes on the Fgf1 Gene. Cell Rep. 2017;18(2):352-66.
4. Tervo DG, Hwang BY, Viswanathan S, Gaj T, Lavzin M, Ritola KD, et al. A Designer AAV Variant Permits Efficient Retrograde Access to Projection Neurons. Neuron. 2016;92(2):372-82.
5. Shumyatsky GP, Malleret G, Shin RM, Takizawa S, Tully K, Tsvetkov E, et al. stathmin, a gene enriched in the amygdala, controls both learned and innate fear. Cell. 2005;123(4):697-709.
6. Shumyatsky GP, Tsvetkov E, Malleret G, Vronskaya S, Hatton M, Hampton L, et al. Identification of a signaling network in lateral nucleus of amygdala important for inhibiting memory specifically related to learned fear. Cell. 2002;111(6):905-18.
7. Martel G, Hevi C, Kane-Goldsmith N, Shumyatsky GP. Zinc transporter ZnT3 is involved in memory dependent on the hippocampus and perirhinal cortex. Behavioural brain research. 2011;223(1):233-8.
8. Martel G, Hevi C, Friebely O, Baybutt T, Shumyatsky GP. Zinc transporter 3 is involved in learned fear and extinction, but not in innate fear. Learning & memory (Cold Spring Harbor, NY. 2010;17(11):582-90.
9. Takao K, Miyakawa T. Light/dark transition test for mice. J Vis Exp. 2006(1):104.

10. Sun YG, Zhao ZQ, Meng XL, Yin J, Liu XY, Chen ZF. Cellular basis of itch sensation. Science (New York, NY. 2009;325(5947):1531-4.
11. Sillivan SE, Joseph NF, Jamieson S, King ML, Chevere-Torres I, Fuentes I, et al. Susceptibility and Resilience to Posttraumatic Stress Disorder-like Behaviors in Inbred Mice. Biological psychiatry. 2017;82(12):924-33.
12. Patriarchi T, Cho JR, Merten K, Howe MW, Marley A, Xiong WH, et al. Ultrafast neuronal imaging of dopamine dynamics with designed genetically encoded sensors. Science (New York, NY. 2018;360(6396).
13. Nieuwenhuis B, Haenzi B, Hilton S, Carnicer-Lombarte A, Hobo B, Verhaagen J, et al. Optimization of adeno-associated viral vector-mediated transduction of the corticospinal tract: comparison of four promoters. Gene Ther. 2021;28(1-2):56-74.
14. Kugler S, Lingor P, Scholl U, Zolotukhin S, Bahr M. Differential transgene expression in brain cells in vivo and in vitro from AAV-2 vectors with small transcriptional control units. Virology. 2003;311(1):89-95.
15. Lerner TN, Shilyansky C, Davidson TJ, Evans KE, Beier KT, Zalocusky KA, et al. Intact-Brain Analyses Reveal Distinct Information Carried by SNc Dopamine Subcircuits. Cell. 2015;162(3):635-47.
16. Pennington ZT, Dong Z, Feng Y, Vetere LM, Page-Harley L, Shuman T, et al. ezTrack: An open-source video analysis pipeline for the investigation of animal behavior. Sci Rep. 2019;9(1):19979.
17. Guarraci FA, Frohardt RJ, Young SL, Kapp BS. A functional role for dopamine transmission in the amygdala during conditioned fear. Annals of the New York Academy of Sciences. 1999;877:732-6.
18. Chen S, Zhou Y, Chen Y, Gu J. fastp: an ultra-fast all-in-one FASTQ preprocessor. Bioinformatics. 2018;34(17):i884-i90.
19. Bray NL, Pimentel H, Melsted P, Pachter L. Near-optimal probabilistic RNA-seq quantification. Nat Biotechnol. 2016;34(5):525-7.
20. Frankish A, Diekhans M, Ferreira AM, Johnson R, Jungreis I, Loveland J, et al. GENCODE reference annotation for the human and mouse genomes. Nucleic Acids Res. 2019;47(D1):D766-D73.
21. R Core Team. R: A Language and Environment for Statistical Computing. Vienna, Austria: R Foundation for Statistical Computing; 2020.
22. Wickham H, Averick M, Bryan J, Chang W, D'Agostino McGowan L, François R, et al. Welcome to the tidyverse. Journal of Open Source Software. 2019;4(43):1686.
23. Soneson C, Love MI, Robinson MD. Differential analyses for RNA-seq: transcript-level estimates improve gene-level inferences. F1000Res. 2015;4:1521.
24. Love MI, Huber W, Anders S. Moderated estimation of fold change and dispersion for RNA-seq data with DESeq2. Genome Biol. 2014;15(12):550.
25. Zhu A, Ibrahim JG, Love MI. Heavy-tailed prior distributions for sequence count data: removing the noise and preserving large differences. Bioinformatics. 2019;35(12):2084-92.
26. Mi H, Muruganujan A, Thomas PD. PANTHER in 2013: modeling the evolution of gene function, and other gene attributes, in the context of phylogenetic trees. Nucleic Acids Res. 2013;41(Database issue):D377-86.
27. Thomas PD, Campbell MJ, Kejariwal A, Mi H, Karlak B, Daverman R, et al. PANTHER: a library of protein families and subfamilies indexed by function. Genome Res. 2003;13(9):2129-41.
